# HexA is a versatile regulator involved in the control of phenotypic heterogeneity of *Photorhabdus luminescens*

**DOI:** 10.1371/journal.pone.0176535

**Published:** 2017-04-27

**Authors:** Angela Langer, Adriana Moldovan, Christian Harmath, Susan A. Joyce, David J. Clarke, Ralf Heermann

**Affiliations:** 1 Bereich Mikrobiologie, Biozentrum Martinsried, Ludwig-Maximilians-Universität München, München, Germany; 2 School of Microbiology and Microbiome Institute, University College Cork, Cork, Ireland; Centre National de la Recherche Scientifique, Aix-Marseille Université, FRANCE

## Abstract

Phenotypic heterogeneity in microbial communities enables genetically identical organisms to behave differently even under the same environmental conditions. *Photorhabdus luminescens*, a bioluminescent Gram-negative bacterium, contains a complex life cycle, which involves a symbiotic interaction with nematodes as well as a pathogenic association with insect larvae. *P*. *luminescens* exists in two distinct phenotypic cell types, designated as the primary (1°) and secondary (2°) cells. The 1° cells are bioluminescent, pigmented and can support nematode growth and development. Individual 1° cells undergo phenotypic switching after prolonged cultivation and convert to 2° cells, which lack the 1° specific phenotypes. The LysR-type regulator HexA has been described as major regulator of this switching process. Here we show that HexA controls phenotypic heterogeneity in a versatile way, directly and indirectly. Expression of *hexA* is enhanced in 2° cells, and the corresponding regulator inhibits 1° specific traits in 2° cells. HexA does not directly affect bioluminescence, a predominant 1° specific phenotype. Since the respective *luxCDABE* operon is repressed at the post-transcriptional level and transcriptional levels of the RNA chaperone gene *hfq* are also enhanced in 2° cells, small regulatory RNAs are presumably involved that are under control of HexA. Another phenotypic trait that is specific for 1° cells is quorum sensing mediated cell clumping. The corresponding *pcfABCDEF* operon could be identified as the first direct target of HexA, since the regulator binds to the *pcfA* promoter region and thereby blocks expression of the target operon. In summary, our data show that HexA fulfills the task as repressor of 1° specific features in 2° cells in a versatile way and gives first insights into the complexity of regulating phenotypic heterogeneity in *Photorhabdus* bacteria.

## Introduction

*Photorhabdus luminescens* is a Gram-negative soil bacterium, which lives in symbiosis with soil nematodes of the genus *Heterorhabditis bacteriophora* and is in turn highly pathogenic against insect larvae [1]. The bacteria colonize the upper gut of the nematodes in its infective juvenile (IJ) stage, which search for insect larvae in the soil. Upon encountering its prey, the nematode enters the hemocoel and releases the bacteria into the insect´s hemolymph by regurgitation [2]. Then, the bacteria produce a huge set of different toxins that effectively kills the insect within 48 hours. Furthermore, the bacteria produce several exoenzymes to convert the cadaver into a rich nutrient soup that is used by the bacteria as well as the nematodes for growth and reproduction. At this stage, the bacteria are bioluminescent and the insect cadaver begins to glow. Antibiotics are produced to defend the cadaver from other bacteria or fungi. When the cadaver is depleted, nematodes in the IJ stage re-associate with the bacteria and search for another insect prey in the soil [3,4].

*P*. *luminescens* exists in two phenotypically different cell types, designated as primary (1°) and secondary cells (2°). The 1° cells are able to associate with the nematodes and show the characteristic features like bioluminescence, pigmentation, production of exoenzymes and antibiotics. The 2° cells lack all these phenotypes [3,5]. In the past, the production of anthraquinones was found to be responsible for the pigmentation of 1° cells. The synthesis of anthraquinones is accomplished via the operon *antABCDEFGHI*, which encodes a type II polyketide synthase and several modifying enzymes [6]. It was recently found, that the regulator AntJ is required for the heterogeneous activation of the expression of *antABCDEFGHI* in 1° cells, whereas only a basal but homogeneous activation was observed in 2° cells. Artificial overproduction of AntJ leads to anthraquininone production in the usually non-pigmented secondary cells, showing that the non-pigmentation is due to a tight regulation of gene expression rather than the result of a special metabolic condition in 2° cells [7].

Despite the fact that 2° cells are also pathogenic towards insect larvae, they are unable to support nematode growth and development. Therefore, it is assumed that 2° cells, although never isolated as free-living form, are better adapted to a free life in the soil as they cannot use the nematodes as a shuttle to reach their prey, like 1° cells do [8]. Furthermore, a huge set of metabolic enzymes was found to be up-regulated in 2° cells, which lends support to the idea that 2° cells have adapted to use the limited nutrients that are present in the soil [9].

Recently, it was found that cell clumping in *P*. *luminescens* is mediated via a novel quorum sensing system. Thereby, so-called photopyrones are produced via the photopyrone synthase PpyS. The photopyrones are recognized by the LuxR solo PluR, which then activates the promoter of the *pcfABCDEF* (*Photorhabdus* clumping factor, PCF) operon. Expression of the *pcf* operon leads to the formation of cell clumps, which is important for the virulence of the bacteria [10]. Whether PCF-mediated cell clumping is another 1° phenotypic feature is unclear.

In the past, the transcriptional regulator HexA has been identified to play an important role in the occurrence of the two phenotypic cell types [11]. In *Photorhabdus temperata* it was previously shown that 1°-specific products are mainly symbiosis factors, which are important for nematode growth and development. These symbiosis factors are repressed in 2° cells by HexA and the derepression of the symbiosis factors in 2° cells resulted in a significant attenuation of virulence to insect larvae. This lent support to the idea that during a normal *Photorhabdus* infection, pathogenicity and symbiosis must be temporally separated and that HexA must be somehow involved in the regulation of this pathogen-symbiont transition [11]. Moreover, HexA has been correlated to control the production of a wide range of different secondary metabolites since a 1° Δ*hexA* mutant drastically increased the production of stilbene-derived small molecules among others, which are known as important symbiosis factors [12]. In a strain where *hexA* is under control of the inducible P_*BAD*_ promoter, the production of mevalagmapeptide, GameXpeptide A, phurealipid A, desmethylphurealipid A as well as photopyrone D was drastically reduced under inducing conditions, revealing that HexA is a global repressor of secondary metabolism in *P*. *luminescens* [13]. HexA belongs to the family of LysR transcriptional regulators (LTTRs) with an N-terminal DNA-binding helix-turn-helix motif and a C-terminal co-inducer-binding domain of yet unknown function. LTTRs belong to the most abundant type of transcriptional regulators of prokaryotes and activators as well as repressors are included in this group [14]. It was found that expression of *hexA* is enhanced in *Photorhabdus temperata* 2° cells. Interruption of *hexA* in 2° cells resulted in a bright phenotype of the normally non-bioluminescent 2° cells. Additionally, the 2° Δ*hexA*::*Tn5* mutant exhibited several other 1° specific phenotypes, further supporting the idea that HexA acts as a repressor of 1° specific phenotypes in the 2° cells [11]. The exact mechanism how HexA can control the various phenotypes remained unclear and DNA-binding of HexA has never been shown. Here we describe that HexA acts as a versatile regulator, which controls gene expression directly at the transcriptional level, and indirectly at the post-transcriptional level and we give first insights into the complexity of the regulation of phenotypic heterogeneity in *P*. *luminescens*.

## Material and methods

### Materials

Strains used in this study are listed in Table A in S1 File, plasmids are listed in Table B in S1 File, and primers are listed in Table C in S1 File. PCRs were performed using Q5 Polymerase and OneTaq Polymerase from New England Biolabs (Frankfurt, Germany). Restriction enzymes and T4 DNA ligase were also purchased from New England Biolabs. Plasmid isolations were performed using the HiYield Plasmid Mini Kit and DNA fragments were purified via the HiYield PCR DNA Fragment Extraction Kit (Süd-Laborbedarf, Gauting, Germany). Genomic DNA was isolated using the Ultra-Clean Microbial DNA Isolation Kit (Mo Bio Laboratories Inc., Carlsbad, USA). Sequencing was performed in the Genomics Service Unit of the LMU Biocenter.

### Bacterial strains and growth conditions

*P*. *luminescens* and *Sh*. *oneidensis* were cultivated aerobically at 30°C and *E*. *coli* was grown aerobically at 37° in lysogenic broth (LB) (10 g NaCl, 10 g/l tryptone, 5 g/l yeast extract) on a rotary shaker. For preparation of agar plates, 1.5% (w/v) agar was added to the medium. If necessary, the medium was supplemented with 50 μg/ml kanamycin, 15 μg/ml gentamicin, 20 μg/ml chloramphenicol, or 100 μg/ml ampicillin. When *E*. *coli* strain ST18 was cultivated, the medium was supplemented with 50 μg/ml 5-aminolevulinic acid.

Pre-cultures were grown overnight and inoculated at an OD_600_ of 0.05 in fresh medium. For induction of the *lac* and the *ara* promoters, different concentrations of IPTG (0.2 mM or 2 mM) and arabinose [0.02% (w/v) or 0.2% (w/v)] were added, respectively unless otherwise stated.

### Generation of the plasmids

For generation of the plasmid pPINT-P_*hexA*_-*hexA*-*mCherry*, P_*hexA*_ and *hexA* were amplified using the primers PhexA-BamHI_fwd and hexA-XmaI_rev using genomic DNA of *P*. *luminescens* TT01 as template. The PCR product was then inserted into the plasmid pPINT-*mCherry* via the restriction sites BamHI and XmaI.

The promoter of *hfq* was amplified by PCR with the primers Phfq-NheI_fwd and Phfq-BamHI_rev using *P*. *luminescens* TT01 genomic DNA as template. The PCR product was cloned into pPINT-*mCherry* using the restriction enzymes NheI and BamHI. For investigating P_*pcfA*_ activity, a PCR with the primers PpcfA-NheI_fwd and PpcfA-XmaI_rev and genomic DNA of *P*. *luminescens* TT01 as template was performed in order to amplify the promoter of *pcfA* with subsequent restriction of the PCR product and pPINT-mCherry with NheI and XmaI. The resulting plasmids pPINT-P_*hfq*_*-mCherry* and pPINT-P_*pcfA*_*-mCherry* were sequenced using the primers check-mcherry-ins_fwd and check-mcherry-ins_rev.

Plasmid pBAD24_P_*ara*_*_pluR_*P_*lac*__*hexA* for *pluR* and *hexA* expression in *E*. *coli* was generated by amplifying the *lacI* gene and the *lac* promoter from the pACYC-Duet1 as template using the primers Plac(h)_fwd and lacI-SalI-rev. The *hexA* gene was amplified from *P*. *luminescens* TT01 genomic DNA template using the primer pair hexA_fwd and hexA-PstI_rev. Subsequently, an overlap PCR was performed by using the primers hexA-PstI_rev and lacI_SalI_rev. The overlap PCR product was ligated into vector pBAD24-*pluR* using restriction enzymes SalI and PstI. Correctness of the resulting plasmid was verified by sequencing using the primers check-Plac-hexA_fwd, pBAD24seq_fwd and hexA_fwd, respectively.

The gene *hexA* was amplified in order to create pBAD24-*hexA* by using the primers hexA-EcoRI_fwd and hexA-NdeI_rev. Subsequently, the PCR product and the plasmid pBAD24-yehU were cut with EcoRI and NdeI and ligated. Correctness of the resulting plasmid was checked by sequencing using the primers pBAD24seq_fwd and pBAD24seq_rev.

In order to generate pBAD24-P_*lac*_-pluR-P_*ara*_-*hexA*, P_*lac*_*-pluR* was amplified with the primers Plac-PluR_fwd and PluR-PstI_rev using pCOLA-*ppyS*-His-*pluR* as a template. The gene *lacI* was amplified using the primers lacI_fwd and lacI-SalI_rev using template pCOLA-*ppyS*-His-*pluR*. An overlap-PCR of *lacI* and P_*lac*_*-pluR* was performed with the primers PluR-PstI_rev and lacI-SalI_rev and inserted into the plasmid pBAD24-hexA via the restriction enzymes PstI and SalI and subsequent ligation. Correct insertion of the DNA fragment was checked by sequencing using the primers pBAD24seq_fwd and pBAD24seq_rev.

Plasmid pACYC_*hexA* was generated by amplifying the *hexA* gene using the primer pair hexA-NcoI_fwd and hexA-SacI_rev and *P*. *luminescens* TT01 genomic DNA as template. The PCR product was then inserted into the vector pACYC via NcoI and SacI restriction sites. The correctness of the plasmid was checked by sequencing using the primers check-pACYC_fwd and check-pACYC_rev.

The plasmid pACYC-P_*lac*_-*hexA*_P_*ara*_-*pluR* was generated in order to place *hexA* under the control of the inducible *lac* promoter and *pluR* under the control of the inducible arabinose promoter for P_*pcfA*_ reporter gene analyses in *Shewanella oneidensis*. Thereby, *araC* and *pluR*, which is under control of the *ara* promoter, were amplified with the primers araCPluR-fwd and pluR-XhoI_rev using pBAD24*-pluR* as template. After restriction with NdeI and XhoI, the PCR product was ligated into equally treated vector pACYC_*hexA*. The correct insertion of the DNA fragment was checked by sequencing using primers pACYC_check_rev and pBAD24seq_fwd.

The plasmid pBBR-P_*lac*_-*lux* was created by amplification of P_*lac*_ from plasmid pEYFP as template with the primers Plac-NheI_fwd and Plac-BamHI_rev, and subsequent ligation via the restriction sites NheI and BamHI and ligation. Correctness of the plasmid was verified by sequencing using the primer check-pBBR-Plac-fwd.

### Generation of the *P*. *luminescens* Δ*hexA* strain

The *hexA* gene was deleted in the *P*. *luminescens* 1° cells using a previously described method [15]. Briefly, the upstream and downstream genomic regions surrounding *hexA* (*plu3090*) were amplified by PCR using the primers FA_hexA_fwd/FA_hexA_rev and FB_hexA_fwd/FB_hexA_rev and the amplicon was cloned into pDS132, resulting in pDS-*hexA*. This plasmid was conjugated from *E*. *coli* S17-1 λ*pir* into 1° cells and exconjugants were selected as Rif^R^ Cm^R^ colonies. The pDS132 plasmid contains the *sacB* gene and, after growth in LB broth (with no selection), putative mutants were identified by screening for Rif^R^ Suc^R^ Cm^S^ colonies. The deletion of *hexA* was confirmed by PCR and DNA sequencing.

### Competent cells and transformations

*E*. *coli* cells were made chemically competent and transformed as described elsewhere [16]. Electrocompetent *E*. *coli* cells were prepared by cultivation of the cells in LB medium at 37°C up to an OD_600nm_ of 1. Cells were then harvested and washed three times with ice-cold 10% (v/v) glycerol and subsequent centrifugation steps (1 min, 16.000 rpm, 4°C). Finally, cells were resuspended in 1/150 of the starting volume in 10% (v/v) glycerol. A similar procedure was used for the preparation of electrocompetent *Sh*. *oneidensis*, except that all washing steps were performed with ice-cold sorbitol (1M). Electrocompetent cells were shortly incubated with 50–100 ng of plasmid DNA and then electroporation was performed in 0.2 cm cuvettes, using a pulse of 2.5 kV for 4–6 msec for *E*. *coli* cells and a pulse of 1.8 kV for 4–6 msec for *Sh*. *oneidensis* cells. The cells were then resuspended in 1 mL LB medium and incubated for 45–60 minutes at 37°C (*E*. *coli)* or 30°C (*Sh*. *oneidensis)* under constant shaking, plated on LB agar supplemented with the appropriate antibiotics and the plates were incubated at 37°C or 30°C overnight.

### Integration of reporter genes into the *P*. *luminescens* genome

For the integration of the different promoter-*mCherry* fusions as well as the *hexA-mCherry* fusion into the genome of *P*. *luminescens*, the donor strain *E*. *coli* ST18 [17], which requires the addition of 5-aminolevulinic acid for growth, was first transformed with the respective plasmids. Then, the conjugative plasmid transfer was achieved via the filter mating method [17]. For that purpose, the donor as well as the recipient strain was cultivated in LB medium at 30° or 37°, respectively, and grown up to an OD_600_ of 0.8–1 in LB medium, which was supplemented with the respective additives if required. The donor strain was washed three times with LB medium and then mixed with the recipient strain in a ratio of 1:5 in a final volume of 1/10 of the donor`s initial volume. The mixed cells were then dropped onto a nitrocellulose filter, which had been positioned into the middle of an LB agar plate. The plate was incubated at 30°C over night, the cells were scratched from the filter and suspended in 500 μl LB medium before they were spread onto LB agar plates containing the appropriate antibiotics. The plates were then incubated for two days at 30°C. Single colonies were picked, cultivated in LB medium at 30°C and the genomic DNA was isolated. Then, the correct insertion of the plasmids into the genome was checked via PCR and sequencing using primers check-rpmE_fwd, oriT_fwd, gmR-pNPTS_fwd, check-mcherry-ins_rev, check-glmS_rev, and the genomic DNA of the clones as template.

### Promoter activity analyses

For promoter activity assays in *E*. *coli* Δ*lrhA and Sh*. *oneidensis*, cells were inoculated in single wells of microtiter plates with an OD_600_ of 0.05 and aerobically grown at 37°C or 30°C, respectively, in a Tecan Infinite F500 plate reader (Tecan, Salzburg) for 8 hours. The OD_600_ and luminescence were measured every 10 minutes. Different concentrations of arabinose and/or IPTG were added to induce *pluR* and/or *hexA* expression after the cells reached an OD_600_ of 0.2. Data are reported as relative light units (RLU) in counts per second per milliliter per OD_600_.

For reporter activity assays in *P*. *luminescens*, the OD_600_ was set to 0.05 and the cells were aerobically grown at 30°C in microtiter plates in a Tecan Infinite F500 system (Tecan, Salzburg). Cells density was determined at OD_600_ and the fluorescence intensity of mCherry was measured (560 nm excitation, 612 nm emission, 20 nm bandwidth). The data were reported as relative fluorescence units (RFU) and then normalized with the optical density (OD_600_) of the respective culture.

### Fluorescence microscopy

In order to investigate promoter activities in *P*. *luminescens* at the single cell level, a fluorescence microscope (Leica, Bensheim) with an excitation wavelength of 546 nm and a 605 nm emission filter with 75 nm bandwidth was used to detect fluorescence of mCherry fluorophore. The respective liquid culture was set to an OD of 0.05 and at appropriate time points, 5 to 15 µl of the culture were dropped onto agarose pads [0.5% (w/v) agarose in PBS buffer, pH 7.4 on a microscope slide]. When cell clumping was investigated, no agar pads were used but the culture was directly dropped onto a microscope slide.

### Two-dimensional gel electrophoresis and protein identification via MALDI-TOF

*P*. *luminescens* TT01-1° and *P*. *luminescens* TT01-1° Δ*hexA* were cultivated aerobically in 200 ml CASO medium at 30°C. After cultivation for 48 hours, cells were incubated with 1 mg/ml (w/v) chloramphenicol to inhibit protein translation and harvested (4.500 x g for 10 min at 4°C). The cells were washed with buffer [100 mM Tris-HCl, pH 7.5, 0.1 mg/ml (w/v) chloramphenicol], and the cell pellets were stored at -80°C until use. After re-suspending the cells in disruption buffer [10 mM Tris-HCl, pH 7.5; 5 mM MgCl_2_; 50 μg/mL (w/v) RNase; 50 μg/mL (w/v) DNase; 100 μg/mL (w/v) lysozyme; 1.39 mM PMSF], they were disrupted by sonification (three times 30 sec pulse interrupted by a 30 sec pause). Cell debris was removed by centrifugation (10 min at 16.100 x g at 4°C). A total protein amount of 350 μg was lyophilized overnight, solubilized in rehydration buffer [8 M urea; 2 M thiourea; 2% (w/v) CHAPS; 1.25% (w/v) IPG buffer pH4-7 l (GE Healthcare, München); 28.4 mM DTT; 0.05% (w/v) bromophenol blue] and separated on IPG stripes (GE Healthcare, München) at 19,650 Vh. The strips were then equilibrated in buffer [50 mM Tris-HCl pH 6.8; 6 M urea; 30% (v/v) glycerol; 4% (w/v) SDS; 18.2 mM DTT] twice for 15 min. Protein separation in the second dimension was performed by SDS-PAGE [18] using 1 mm gels of the size 16 cm × 20 cm with 13% (w/v) acrylamide in the separation gel and 7% (w/v) in the spacer gel at 15°C with 5 mA overnight. Gels were stained for 1 h in Coomassie blue solution [40% (v/v) ethanol; 10% (v/v) acetic acid; 0.2% (w/v) Coomassie brilliant blue R250], destained for 1 h with destaining solution [40% (v/v) ethanol; 10% (v/v) acetate], and the entire complete destaining was performed with 10% (v/v) acetic acid (Weber K, 1969). The gels were scanned and analyzed via the PDQuest software (Bio-Rad, München) in triplicates, comparing the wild-type TT01-1°proteome with the proteome of TT01-1°Δ*hexA*. Proteins with altered production pattern were analyzed by MALDI-TOF. For that purpose, protein spots were cut out of the gels, washed with deionized water (four times for 30 min at 37°C while shaking at 850 rpm) and incubated in 50% (v/v) acetonitrile twice for 15 min at 37°C. Proteins were then digested via the addition of trypsin in 40 mM ammonium bicarbonate (Promega, Mannheim) overnight at 37°C and samples were desalted using ZipTip μ-C18 columns (Millipore, Eschborn), and then directly eluted with 1 μl matrix [saturated solution of α-cyano-4-hydroxy-cinnamic acid in 50% (v/v) acetonitrile and 0.6% (v/v) trifluoroacetic acid]. The samples were analyzed in a Voyager DE STR MALDI-TOF system (Applied Biosystems, Foster City, USA) using the reverse modus in the range of 700–3.500 Da. Peptide masses were calibrated using known masses of autolysis peptide of trypsin. Proteins were identified via their masses using the search engine MASCOT on http://www.matrixscience.com/ [19].

### Purification of HexA-6His

HexA-6His was purified using Ni^2+^-NTA affinity chromatography. As first step, *E*. *coli* BL21 cells harboring plasmid pBAD24-*hexA* were cultivated up to an OD_600_ of 0.5 and gene expression was induced by adding 0.1% (w/v) L-arabinose. After 4 h of aerobic cultivation at 30°C, cells were harvested and disrupted using a cell disruptor (Constant Cell Disruption Systems, Northants, UK) at 1.35 kbar and 4°C in lysis buffer [50 mM Tris-HCl (pH 7.5), 10% glycerol (v/v), 10 mM MgCl_2_, 0.5 mM PMSF, 1 mM DTT and 10 ng/mL DNase I]. Subsequently, HexA was eluted using elution buffer B [50 mM Tris-HCl (pH 7.5), 10% (v/v) glycerol, 200 mM NaCl, 250 mM imidazole, 2 mM β-mercaptoethanol]. Purification technique was in principle carried out as described for the response regulator KdpE before [20]. The equilibration and washing steps were performed in buffer E [50 mM Tris-HCl (pH 7.5), 10% (v/v) glycerol, 200 mM NaCl, 2 mM β-mercaptoethanol, 10 mM imidazole] and 250 mM imidazole (final concentration) was added for the elution of the protein from the column. Purified HexA was subjected to SDS-PAGE and the bands of eight lanes (approximately 100 μg protein) corresponding to HexA were cut out of the gel and used to generate αHexA rabbit IgG antiserum at BioGenes GmbH (Berlin, Germany).

### Surface plasmon resonance (SPR) spectroscopy

SPR assays were performed in a Biacore T200 (GE Healthcare, München) using carboxymethyl dextran sensor chips pre-coated with streptavidin (XanTec SAD500L, XanTec Bioanalytics GmbH, Düsseldorf). Biotinylated fragments of the *pcfA* promoter were generated by amplification of the P_*pcfA*_ region using chromosomal DNA of *P*. *luminescens* as template with the biotinylated primer PpcfA-Btn_fwd and PpcfA _rev. As a negative control a *sacB* fragment was amplified using the biotinylated primer sacB-Btn_fwd and sacB_rev. Before immobilizing the DNA fragment, the chip was equilibrated by three injections using 1 M NaCl/50 mM NaOH at a flow rate of 10 μl/min. 10 nM of the respective double-stranded biotinylated DNA fragment was injected using a contact time of 420 seconds and a flow rate of 10 μl/min. 1 M NaCl/50 mM NaOH/50% (v/v) isopropanol was injected as a final wash step. Approximately 300 RU of the DNA fragment was captured onto flow cell 2 or 4, respectively, of the chip. HexA was diluted in HBS-EP+ buffer [10 mM HEPES, pH 7.4, 150 mM NaCl, 3 mM EDTA, 0.05% (v/v) Surfactant P20] and passed over flow cells 1 to 4 in different concentrations (0 nM, 125 nM, 250 nM, 500 nM, 1000 nM and 2000 nM) using a contact time of 180 sec followed by a 240 sec dissociation time before the next cycle started. The experiments were carried out at 25°C at a flow rate of 30 μl/min. After each cycle, regeneration of the surface was achieved by injection of 2.5 M NaCl for 60 sec at 30 μl/min flow rate. Sensorgrams were recorded using the Biacore T200 Control software 2.0 and analyzed with the Biacore T200 Evaluation software 2.0 (GE Healthcare, München). The surface of flow cell 1 was used to obtain blank sensorgrams for subtraction of bulk refractive index background. The referenced sensorgrams were normalized to a baseline of 0. The 1:1 binding algorithm was used for calculation of the binding affinity. Peaks in the sensorgrams at the beginning and the end of the injection emerged from the runtime difference between the flow cells of each chip. Experiments were performed in the Bioanalytics core facility of the LMU München.

### Fluorescence-based thermal stability assay

The fluorescence-based thermal stability assays were performed as described before [21]. Purified HexA-6His was used at a final concentration of 0.72 μM. Various buffers containing different concentrations of glycerol, β-mercaptoethanol and urea were tested. The iQ5 real-time PCR detection system (BioRad, München) with a temperature gradient of 1°C/ min from 5°C to 95°C was used. Results were obtained from three independently performed experiments.

## Results

### *P*. *luminescens* 2° cells contain increased levels of HexA

**Fig 1:**
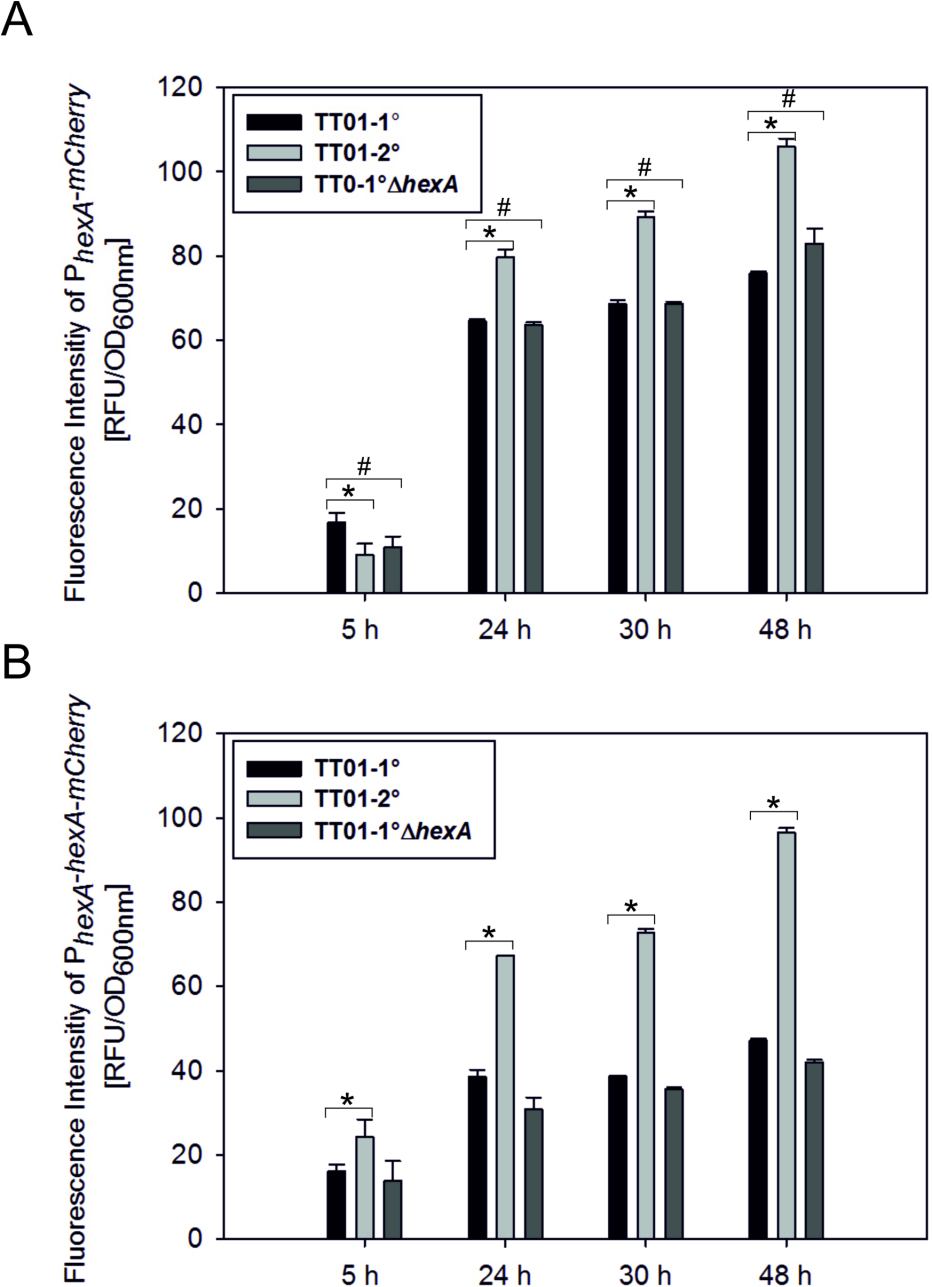
Transcriptional and translational levels of *hexA* expression. The transcriptional level, by fusing the promoter of *hexA* to *mCherry* (A), as well as the translational level, by generating protein hybrids of HexA with mCherry under the control of the *hexA* promoter (B), were investigated. The reporter constructs P_*hexA*_*-mCherry* and P_*hexA*_-*hexA-mCherry*, respectively, were integrated into the chromosome and fluorescence intensities were measured after 5 h (early exponential phase), 24 h (mid-exponential phase), 30 h (stationary phase), and 48 h (late stationary phase) in the *P*. *luminescens* strains TT01-1°, TT01-2°, TT01-1°Δ*hexA*. The asterisk (*) indicates statistically significant differences with a p-value smaller than 0.05. The hash (#) indicates no statistically significant differences with a p-value bigger than 0.05. Error bars represent standard deviation of three independently performed experiments.

As a first step to get more insights into the mechanism of HexA to act as a regulator of phenotypic heterogeneity of *P*. *luminescens*, we investigated HexA levels in 1° and 2° cells. In previous studies, via Northern Blot analyses it was shown that the *hexA* mRNA levels in different *Photorhabdus* species are enhanced in 2° compared to 1° cells [11]. To further investigate HexA levels in *P*. *luminescens*, we determined the promoter activity of *hexA* using strains carrying a chromosomal P_*hexA*_*-mCherry* fusion in a 1°, 2° as well as in the 1°Δ*hexA* background and measured the fluorescence after 5 h, 24 h, 30 h, and 48 h of growth [22]. The fluorescence intensities were up to 1.4 fold higher in 2° cells compared to 1° cells at time point 48 h (p-value < 0.05). The fluorescence was comparable in a 1° *hexA* deletion strain and 1° wild-type cells at all time points (p-value >0.05), revealing that P_*hexA*_ activity is not under autoregulation of HexA (Fig 1A). As a next step, we generated a chromosomal translational fusion of P_*hexA-*_*hexA*-*mCherry* and determined the reporter gene activity in the *P*. *luminescens* 1°, 2° as well as in the 1°Δ*hexA* background after 5 h, 24 h, 30 h, and 48 h of growth. Fluorescence intensities were approximately two-fold higher in 2° compared to 1° cells after 24 h of growth (p-value < 0.05), revealing that not only *hexA* transcription, but also HexA protein levels are enhanced in 2° cells (Fig 1B). Moreover, P_*hexA-*_*mCherry* as well as P_*hexA-*_*hexA*-*mCherry* mediated fluorescence was homogeneously distributed 1° as well as in 2° cells at the single cell level (Figure A in S1 File). Therefore, our data confirms previous reports describing enhanced HexA levels in *P*. *temperata* and *P*. *luminescens* 2° cells [11].

### Influence of HexA on the 1° specific phenotype bioluminescence

**Fig 2:**
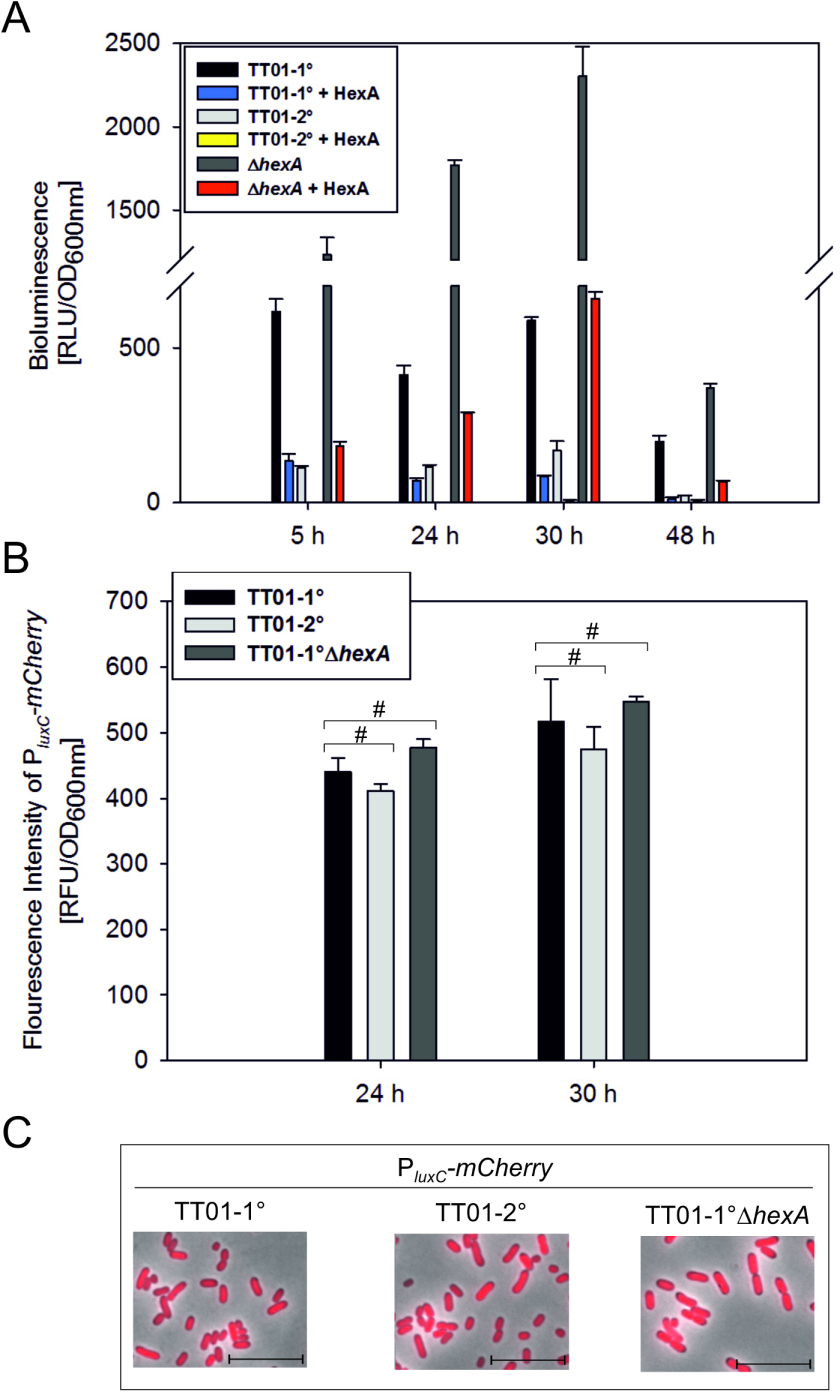
Effect of HexA on the bioluminescence of *P*. *luminescens*. Bioluminescence in *P*. *luminescens* TT01-1°, TT01-2° and TT01-1°Δ*hexA* under native conditions and with enhanced levels of HexA. Error bars represent standard deviation of three independently performed experiments (A). Promoter activity of *luxCDABE* at the population level. The respective reporter construct P_*luxC*_*-mCherry* was integrated into the chromosome and fluorescence intensities were measured after 24 h (mid-exponential growth phase) and 30 h (stationary growth phase) in the strains TT01-1°, TT01-2°, TT01-1°-Δ*hexA*. The hash (#) indicates no statistically significant differences with a p-value bigger than 0.05. (B). P_*luxC*_ activity in strains TT01-1°, TT01-2° and TT01-1°Δ*hexA* at the single cell level. The scale depicts 10 μm. Representative images from one of three independently performed experiments are shown (C).

One of the most striking phenotypic differences between 1° and 2° cells of *P*. *luminescens* is bioluminescence, which is predominantly present in 1° cells. Since the studies with the P_*hexA*_*-mCherry* and P_*hexA*_*-hexA*-*mCherry* reporters revealed a putative involvement of HexA in the regulation of 1° specific phenotypes, we investigated a potential influence of HexA on bioluminescence by comparing light production of a 1°, 2° and 1°Δ*hexA* population during growth. We observed that the bioluminescence is significantly increased in the 1°Δ*hexA* population, especially when the cells enter the stationary growth phase (Fig 2A). It was hypothesized before that HexA might act as repressor for bioluminescence in 1° cells [11]. As expected, light production is only weakly detectable in the 2° population in all growth phases. When introducing only one additional copy of *hexA* that is under control of its native promoter the bioluminescence drastically decreased in the 1° cells. Furthermore, introduction of an additional *hexA* gene completely abolished bioluminescence in the 2° population. In the stationary growth phase, enhanced HexA levels led to a decrease in bioluminescence of 86% in the 1° population. Complementation of *hexA* in the 1°Δ*hexA* population reduced the production of light to its native 1° level. In summary, *hexA* gene dosage and the resulting level of HexA is crucial for the different levels of light production in 1° and 2° cells.

Bioluminescence is, in contrast to other 1° specific phenotypes such as antibiotic or exoenzyme production, correlated with the expression of a single operon, which makes it easy to correlate this phenotype with the respective promoter activity. We therefore investigated the influence of HexA on the expression of the corresponding *luxCDABE* operon. For that purpose, we fused the P_*luxC*_ promoter to *mCherry* and integrated a single copy of the reporter into the chromosome of *P*. *luminescens* 1°, 2° and 1°Δ*hexA*. However, the fluorescence intensities of all three reporter strains and therefore the P_*luxC*_ activity was comparable at the population level (p-value > 0.05), even though the respective bioluminescence phenotypes were completely different (Fig 2B). Furthermore, the P_*luxC*_ activity was homogeneously distributed in all three reporter strains at the single cell level (Fig 2C). Thus, HexA does not regulate transcription of the *luxCDABE* operon, but rather regulates light production at the post-transcriptional level.

### Hfq is involved in HexA regulation

**Fig 3:**
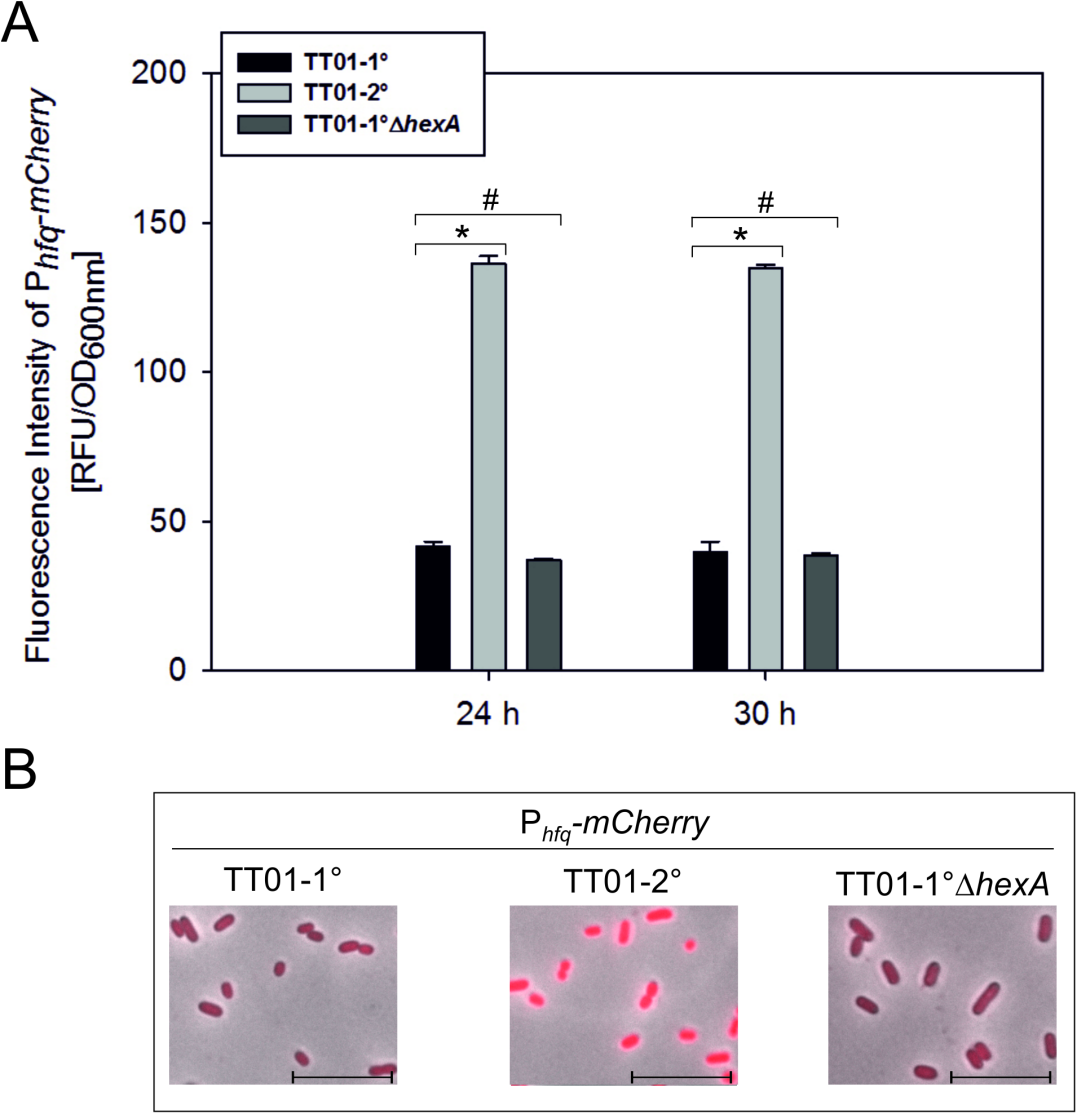
Transcriptional levels of *hfq* in *P*. *luminescens* TT01-1°, TT01-2° and TT01-1°Δ*hexA*. Promoter activity of *hfq* at the population level. The respective reporter construct P_*hfq*_*-mCherry* was integrated into the chromosome and fluorescence intensities were measured after 24 h (mid-exponential phase) and 30 h (stationary phase) in the strains TT01-1°, TT01-2°, TT01-1°Δ*hexA*. The asterisk (*) indicates statistically significant differences with a p-value smaller than 0.05. The hash (#) indicates no statistically significant differences with a p-value bigger than 0.05. Error bars represent standard deviation of three independently performed experiments (A). P_*hfq*_ activity in TT01-1°, TT01-2° and TT01-1°Δ*hexA* at the single cell level. The scale depicts 10 μm. Representative images from one of three independently performed experiments at time point 48 h are shown (B).

Regulation of gene expression at the post-transcriptional level is often controlled by small regulatory RNAs (sRNAs) mediated by the RNA chaperon Hfq (see [23] for overview). Therefore, we investigated whether Hfq is also involved in the regulation of phenotypic heterogeneity in *P*. *luminescens*. We first introduced a chromosomal copy of P_*hfq*_*-mCherry* into 1°, 2° and 1°Δ*hexA* cells and investigated the fluorescence intensities and therefore P_*hfq*_ activities of all reporter strains at different growth phases. The 1°Δ*hexA* population showed P_*hfq*_ activities comparable to the 1° population (p-value > 0.05), except for the early exponential growth phase where it was slightly enhanced. However, the P_*hfq*_ activity was up to 3.4-fold enhanced (p-value < 0.05) in the 2° compared to the 1° population (Fig 3A). The promoter activity of P_*hfq*_ was homogeneously distributed in all three reporter strains (Fig 3B). This reveals that Hfq and therefore presumably regulation via sRNAs may be involved in the regulation of phenotypic heterogeneity in *P*. *luminescens*.

### Influence of HexA on the primary specific feature cell clumping

**Fig 4:**
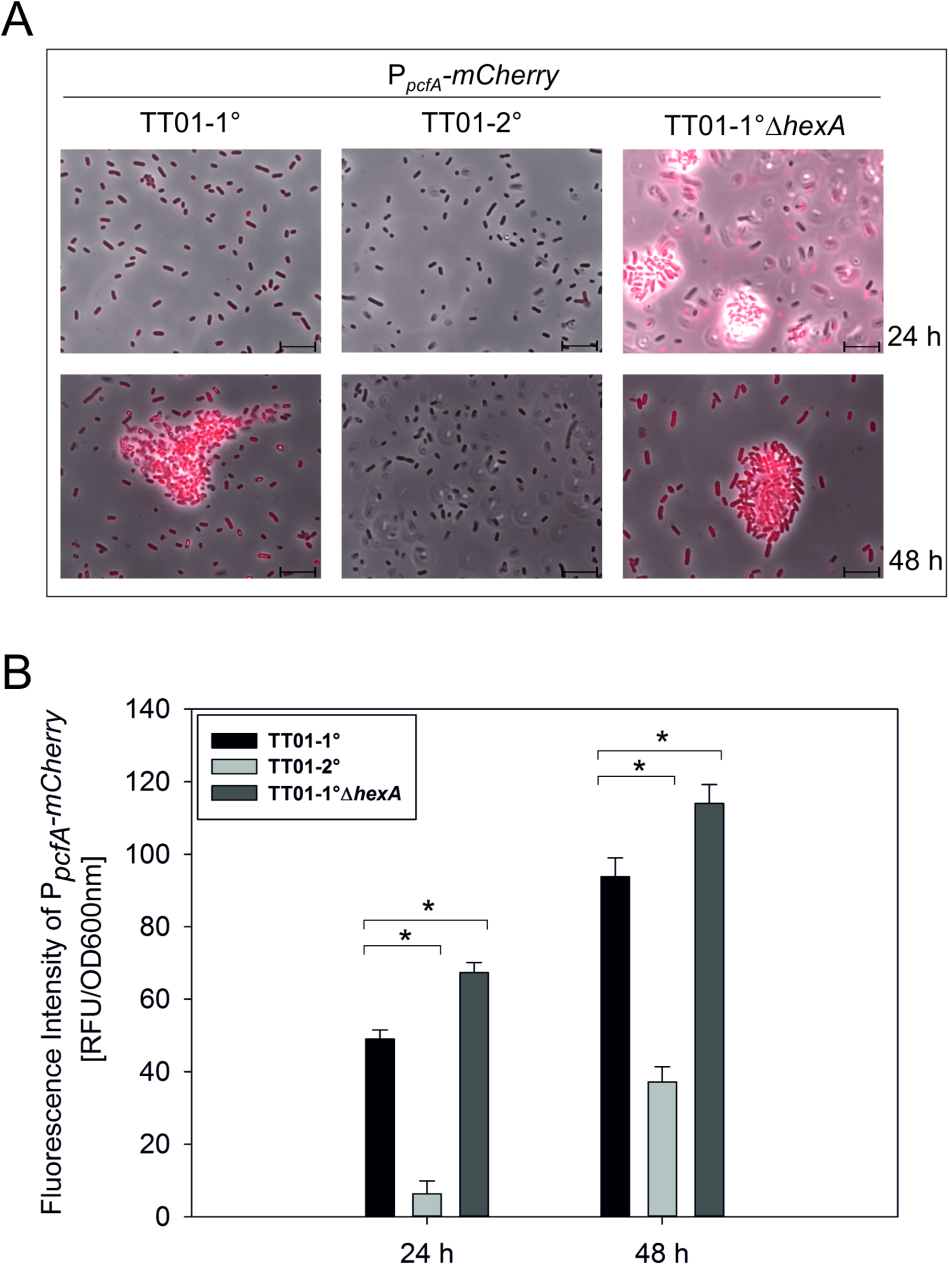
Cell clumping in *P*. *luminescens* TT01-1°, TT01-2° and TT01-1°*ΔhexA*. P_*pcfA*_*-mCherry* activity and cell clumping in TT01-1°, TT01-2° and TT01-1°Δ*hexA*. The scale depicts 10 μM. Representative images from one of three independently performed experiments are shown (A). Promoter activity of *pcfABCDEF* at the population level in TT01-1°, TT01-2° and TT01-1°Δ*hexA*. The asterisk (*) indicates statistically significant differences with a p-value smaller than 0.05. Error bars represent standard deviation of three independently performed experiments (B).

In order to investigate the full regulatory influence of HexA we compared the proteomes of the exponential and stationary growth phase of 1° and 1°Δ*hexA* cells via 2D-PAGE, and identified 22 proteins that were differentially produced in the *hexA* deletion strain (Figure B and Table D in in S1 File). These included proteins that are involved in metabolism, antibiotic and toxin production, cell adhesion, three proteins of yet unknown function and one regulator (PAS4-LuxR receptor, Plu2016). Furthermore, elevated levels of the three proteins PcfA, PcfB and PcfC were found in the Δ*hexA* strain, which are encoded by the *pcfABCDEF* (*Photorhabdus* clumping factor) operon, responsible for the formation of cell clumps in *Photorhabdus* and regulated by the novel PpyS/PluR quorum sensing system [10]. To determine if HexA is directly involved in regulation of *pcfABCDEF* expression, and therefore cell clumping, we integrated a P_*pcfA*_-*mCherry* reporter into the genomes of *P*. *luminescens* 1°, 2° and 1°Δ*hexA* and then analyzed fluorescence intensities as well as cell clumping at different growth phases via microscopy. Clumps of 1° cells were visible at the beginning of the stationary growth phase (Fig 4A, 48 h of incubation). The 1°Δ*hexA* strain already formed cell clumps in the exponential growth phase (Fig 4A, 24 h of incubation). However, the 2° cells did not form cell clumps, even after seven days of incubation (Fig 4A, Figure C in S1 File). Furthermore, the activity of P_*pcfA*_ was extremely high in the 1°Δ*hexA* mutant compared to the 1° cells (p-value < 0.05) and totally absent in 2° cells (Fig 4B). Therefore, these results strongly suggest that HexA acts as a repressor of the *pcf* operon.

### Binding of HexA to the P_*pcfA*_ promoter

**Fig 5:**
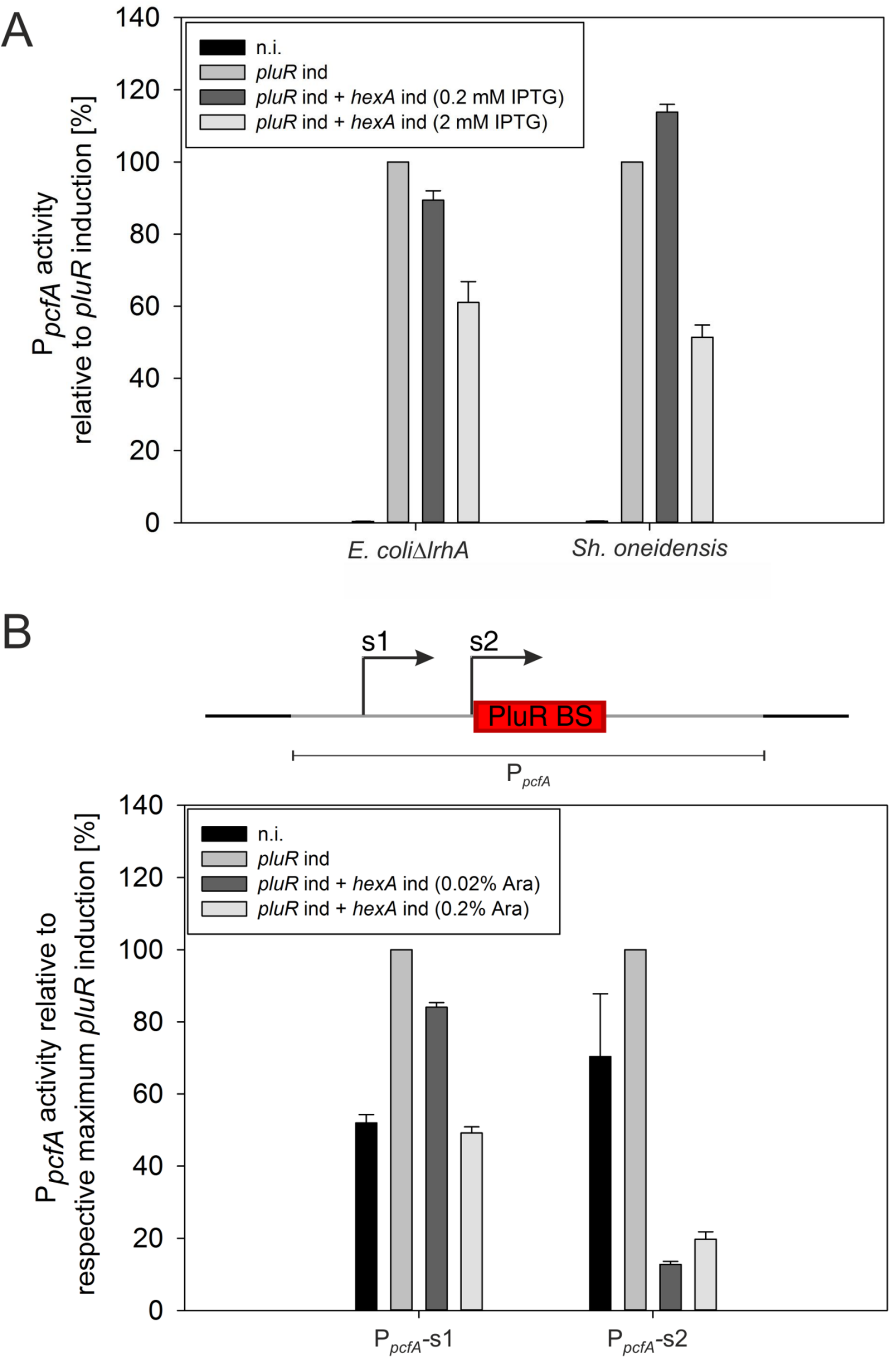
Effect of HexA on the P_*pcfA*_ activity in the heterologous systems of *E*. *coli* Δ*lrhA* and *Sh*. *oneidensis*. *E*. *coli* Δ*lrhA and Sh*. *oneidensis* were transformed with plasmids pBAD24-P_*ara*_-*pluR*_P_*lac*_-*hexA* and pACYC-P_*lac*_-*hexA*_P_*ara*_-*pluR*, respectively, in combination with plasmid pBBR-P_*pcfA*_*-lux*. In *E*. *coli* Δ*lrhA* the *pluR* expression was achieved via the addition of 0.1% (w/v) arabinose and in *Sh*. *oneidensis* 0.02% (w/v) arabinose was added for *pluR* expression. The values were measured as relative light units [RLU] divided by OD_600nm_ (A). In the upper panel the promoter region of *pcfA* with the PluR binding site (PluR BS) is depicted. In *E*. *coli* Δ*lrhA* two different truncations s1 and s2 of the *pcfA* promoter were tested. Thereby, *pluR* induction was achieved via the addition of 1 mM IPTG and *hexA* expression was induced via 0.02% (w/v) or 0.2% arabinose (w/v) on plasmid pBAD24-P_*lac*_-*pluR*_P_*ara*_-*hexA* (B). The figures represent three biological replicates; n.i.: non-induced, ind: induced; All values are given in percentage, relative to the respective maximum *pluR* induction.

To investigate whether HexA directly or indirectly regulates expression of *pcfABCDEF*, we determined P_*pcfA*_ activity in *E*. *coli* as a heterologous system. Since *E*. *coli* contains a HexA homolog called LrhA, an *E*. *coli* Δ*lrhA* strain was used and the expression of the *luxCDABE* operon was put under the control of the *pcfA* promoter. P_*pcfA*_ activity can be induced by over-expression of *pluR* since PluR is a direct activator of *pcfABCDEF* expression [10,24]. For that reason, the reporter strain was further equipped with *pluR*, which is under control of the arabinose inducible promoter P_*ara*_. The *hexA* gene was set under control of the P_*lac*_ promoter and its expression could therefore be achieved via the addition of IPTG. Thus, the addition of arabinose led to a signal-independent activation of P_*pcfA*_ by simple overproduction of PluR. We observed that with increasing concentrations of IPTG and thus with increasing levels of HexA, the activity of P_*pcfA*_ decreased to 61% (Fig 5A). These results strongly suggest that HexA represses *pcfABCDEF* expression. Since *E*. *coli* contains the LysR-type regulator and HexA homolog LrhA, the repression in this heterologous system might still be indirect although LrhA is absent. In order to further exclude any influence of LrhA, we also tested the similar P_*pcfA*_ reporter assay in *Shewanella oneidensis*, which lacks any homolog of HexA. A BLAST analysis of HexA on the *Sh*. *oneidensis* proteome showed that the most similar protein to HexA is the LysR-type regulator SO_997, with only 32% identity and 47% homology (compared to 57% identity and 77% homology of LrhA of *E*. *coli* to HexA). Similar to the observations in the *E*. *coli* reporter strain, HexA overproduction also led to a decrease of P_*pcfA*_ activity, which dropped to 49% compared to the conditions where HexA was not overproduced (Fig 5A). To confirm that the results were not due to differences in the chosen inducible promoters P_*ara*_ and P_*lac*_, the promoters were swapped, putting *pluR* expression under the control of P_*lac*_ and *hexA* expression under the control of P_*ara*_. Comparable results as described above were obtained (Figure D in S1 File). To exclude potential effects of HexA on the *luxCDABE* operon, a reporter assay using P_*lac*_-*luxCDABE* was performed in *E*. *coli* Δ*lrhA*. No inhibiting effect due to HexA overproduction was detected (Figure E in S1 File). In summary, these data support the idea that HexA directly controls the activity of P_*pcfA*_.

The binding site of PluR within the P_*pcfA*_ region was identified (Sophie Brameyer and Ralf Heermann, LMU, unpublished information), which is located downstream of the S2 site (Fig 5B). We were now interested to determine whether the binding site of HexA is located up- or downstream of the PluR binding site. Therefore, we tested the activity of different truncated *pcfA* promoter constructs for inhibition by HexA using the above-described heterologous *E*. *coli* Δ*lrhA* P_*pcfA*_-*luxCDABE* reporter system. When the promoter upstream of the PluR binding site was deleted (S1), the HexA-mediated repression could still be observed as P_*pcfA*_ activity was reduced by 50% upon HexA overproduction (Fig 5B). Using a P_*pcfA*_-construct truncated close to the PluR binding site (S2), a reduced activation via PluR was measured (data not shown). Nevertheless, compared to the respective maximum induction of this promoter construct, a HexA-mediated repression of 80% was observed (Fig 5B). Thus, it is assumed that HexA binds downstream of the PluR binding site. However, this could not be investigated further using this assay as any additional truncation would destroy the PluR DNA-binding site and P_*pcfA*_ could not be activated any more.

**Fig 6:**
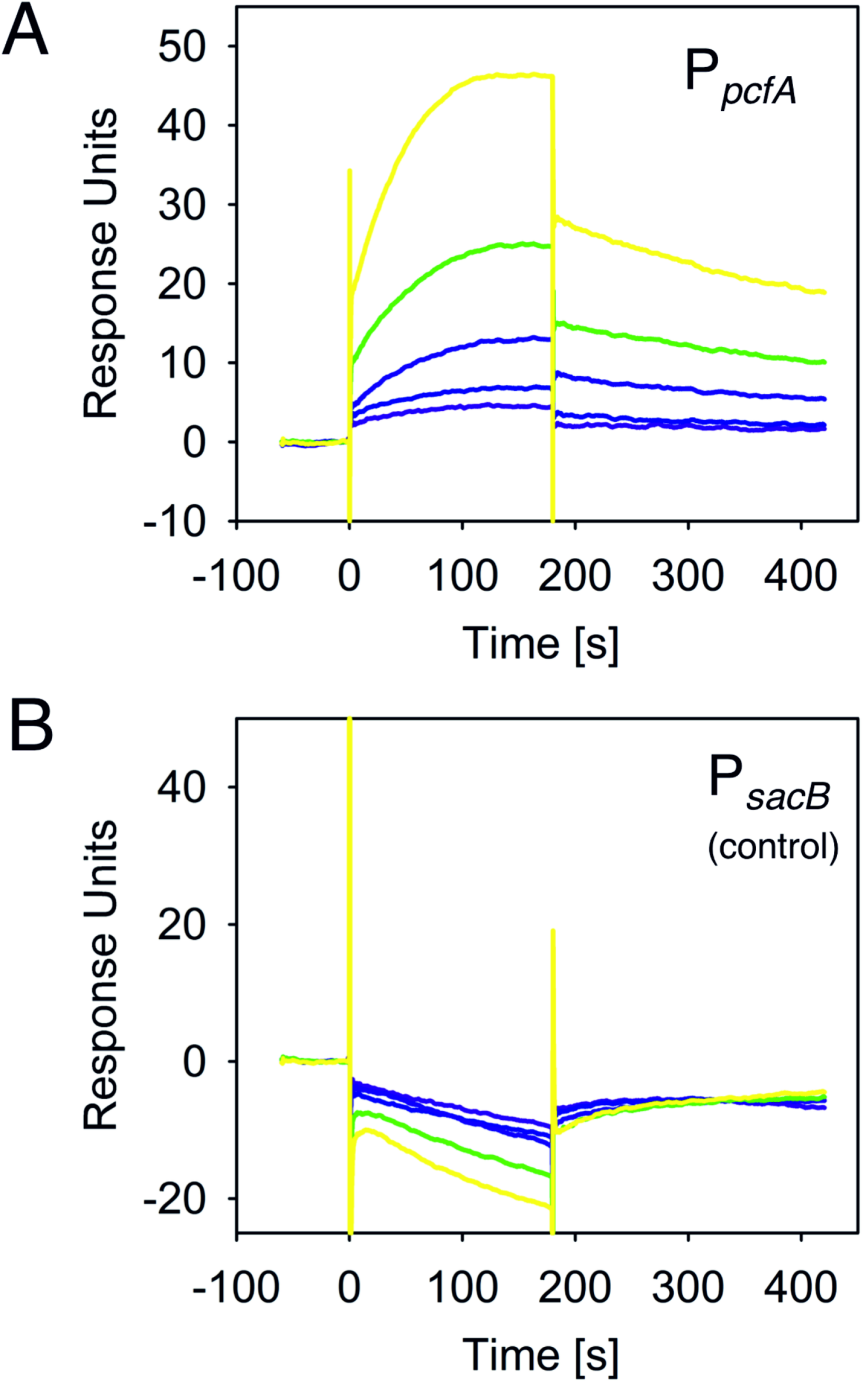
Direct binding of HexA to the *pcfA* promoter region. The biotinylated P_*pcfA*_ DNA-fragment was captured onto a streptavidin-coated (SA) sensor-chip. Different concentrations of His-tagged HexA (125 nM: purple line; 250 nM: dark blue line; 500 nM: light blue line; 1000 nM: green line; 2000 nM: yellow line) were passed over the chip. An overall affinity of K_D_ 1.3 μM was determined, the association and dissociation rates were determined as k_a_ = 1300 M*s and k_d_ = 0.002 1/s, respectively. As a negative control for unspecific binding, the SA chip was coated with a *sacB* DNA fragment.

To prove direct binding of HexA to the *pcfA* promoter region, we performed surface plasmon resonance (SPR) spectroscopy. As a first step, a HexA-6His variant was overproduced and purified using Ni-NTA affinity chromatography. The purification of the correct protein was verified using αHexA specific antibodies. Then, optimal buffer conditions were identified via a fluorescence-based thermal stability assay screen. Gel filtration experiments as well as dynamic light scattering assays showed that HexA is primarily present as a tetramer (Figure F in S1 File). For the SPR assays, a 400 bp biotinylated DNA fragment comprising the *pcfA* promoter region was captured onto a streptavidin pre-coated sensor chip. As a control, a 400 bp DNA fragment of the *sacB* gene from *Bacillus subtilis* was used. Subsequently, different concentrations of purified HexA were injected and passed over the chip surface. As can be seen in Fig 6, HexA specifically bound to the *pcfA* promoter DNA with an overall affinity (K_D_) of 1.3 μM, which was calculated by the 1:1 binding algorithm. However, the sensorgram shape does not reveal a true 1:1 binding, since even at higher HexA concentrations the sensorgrams do not reach saturation. Due to the low association (*k*_*a*_ = 1300/M*s) and very high dissociation (*k*_*d*_ = 0.002/s) it appears likely that a potential HexA ligand is absent and this ligand might influence the DNA-binding affinity of HexA. However, the addition of several primary metabolites (amino acids, sugars, compounds of the tricarboxylic acid cycle) and also secondary metabolites (cinnamic acid) did not influence the DNA-binding activity of HexA. However, although a putative ligand of HexA that mediates DNA-binding activity remains elusive, we have shown for the first time that HexA is a DNA-binding protein. Moreover, HexA exerts its regulation on phenotypic variation using a combination of direct (e.g. *pcf* operon) and indirect (e.g. *lux* operon) mechanisms.

## Discussion

The LysR-type regulator HexA is a major regulator in control of phenotypic heterogeneity in *P*. *luminescens*. It has been previously proposed that HexA might act as a global repressor of 1° specific genes in *P*. *temperata* [11], but it was unclear how HexA could fulfill the function as a direct regulator of such a huge subset of genes and operons that are specifically expressed in 1° and not in 2° cells. Here we show for the first time that HexA is a regulator that can bind DNA and therefore acts as direct repressor of 1° specific genes like cell clumping in *P*. *luminescens*. Moreover, HexA can also indirectly (probably using posttranscriptional mechanisms) influence the expression of 1° specific genes such as the *lux* operon. It is likely that sRNAs are involved in this process since the promoter activity of *hfq*, encoding for the RNA chaperone Hfq, is enhanced in 2° cells. We could confirm increased transcriptional levels of *hexA* in *P*. *luminescens* 2° cells, supporting the assumption that HexA acts as repressor of 1° specific features. In the early exponential phase, the transcriptional as well as the translational fusions of *hexA* with *mCherry* were comparable between 1° and 2° cells, and the biggest differences were detectable when the cells entered the stationary growth phase. These results support the fact that HexA mainly regulates the phenotypic switching process in the stationary phase since the 1° specific features typically occur in the post-exponential growth phase. Furthermore, the translational fusions of P_*hexA*_*-hexA-mCherry* clearly showed that the 2° cells have higher HexA protein levels than the 1° cells. The translational reporter displayed even stronger differences between 1° and 2° cells than those observed using the transcriptional fusions (P_*hexA*_*-mCherry*). This suggests that the level of HexA protein is regulated at the transcriptional and the posttranscriptional level, e.g. less degradation of mature proteins leading to a longer half-life of HexA in 2° cells. This is in accordance with the identification of a protease inhibitor in 2° cells [25]. *E*. *coli* contains a homologous regulator of HexA, which is called LrhA that is known to be involved in the repression of flagella, motility and chemotaxis genes [26]. In contrast to HexA of *P*. *luminescens*, LrhA of *E*. *coli* positively auto-regulates expression of its own gene [26]. However, we could not identify any auto-regulation of *hexA* in *P*. *luminescens*. In closely related entomopathogenic *Xenorhabdus nematophila*, *lrhA* expression was found to be under control of the CpxA/CpxR two-component system [27], which detects cell envelope stress [28]. Therefore, *hexA* expression in *P*. *luminescens* may also be fine-tuned by a two-component system like CpxA/CpxR and might therefore be somehow connected to environmental stress.

How does HexA indirectly influence expression of 1° specific genes? We investigated the 1° specific feature bioluminescence and could show, that light production is dependent on HexA in *P*. *luminescens*. The deletion of *hexA* led to the brightest phenotype and complementation of *hexA* restored the native levels of bioluminescence. In contrast, promoter activity of the *luxCDABE* operon showed no significant differences in 1°, 2° and 1°Δ*hexA* cells, suggesting a post-transcriptional regulation of *luxCDABE* expression. In *E*. *carotovora* as well as in *E*. *coli*, it was found that HexA or LrhA, respectively, control the levels of RpoS and therefore influence the expression of hundreds of stationary-phase genes [29,30]. Furthermore, in *E*. *coli* it could be found that LrhA represses the sRNA RprA, which is an activator of RpoS translation. Another unidentified sRNA, influencing RpoS translation in an RprA-independent manner, is regulated by LrhA [31]. The absence of the stationary-phase specific features in 2° cells of *P*. *luminescens* might also be explained by reduced RpoS levels caused by enhanced HexA levels. However, as LrhA in *E*. *coli* is known to regulate sRNAs it seems even more likely that HexA in *P*. *luminescens* also influences the expression of sRNAs to control 1° specific gene expression. This is supported by the fact that the promoter activity of *hfq* is enhanced in 2° cells. Hfq is an RNA chaperone that facilitates RNA-RNA interactions involved in post-transcriptional regulation [32]. Hfq is known to be required for the stability and/or interactions with the target mRNA of numerous *E*. *coli* sRNAs [33]. It is known that several sRNAs *e*.*g*. DsrA. OxyS and RprA are involved in regulation of *rpoS* translation. Interestingly, in turn the association with Hfq is needed for the function of these sRNAs. Furthermore, it is described that LrhA-dependent repression of *rpoS* translation is also dependent on the RNA chaperone Hfq in *E*. *coli* [31]. We could not detect any differences of *hfq* promoter activity upon deletion of *hexA* in 1° cells. Therefore, we conclude that Hfq influences *hexA* expression and not *vice versa*. This is supported by the finding that a deletion of *hfq* in *P*. *luminescens* 1° cells caused a drastic increase in *hexA* expression [34]. Furthermore, nearly no production of secondary metabolites and the inability to support symbiosis with nematodes was observed for the 1°Δ*hfq* mutant. A double deletion of *hfq* and *hexA* restored secondary metabolite production as well as the recovery of infective juveniles to nematodes [34]. However, we observed enhanced HexA levels and increased promoter activity of *hfq* in 2° cells. Since Hfq is known to be autoregulated at the translational level in *E*. *coli* [35], it could also be possible that translation of *hfq* or protein activity is somehow diminished or impaired in *P*. *luminescens* 2° cells.

We found that HexA directly regulates expression of the *pcfABCDEF* operon, which induces cells clumping. The promoter activity of *pcfA* was repressed in 2° cells and enhanced upon deletion of *hexA* in 1° cells, suggesting that HexA represses *pcfABCDEF* expression. Direct binding to the *pcfA* promoter region was verified by SPR spectroscopy. The *pcf* operon is under positive control of the PpyS/PluR quorum sensing system in *P*. *luminescens* [10]. Thereby, *P*. *luminescens* does not communicate via acyl-homoserine-lactones (AHLs) like many other Gram-negative bacteria, but by photopyrones (PPYs), which are produced by the photopyrone synthase PpyS and sensed by the LuxR-type receptor PluR. We found that the formation of cell clumps is impaired and the promoter activity of the *pcf* operon is repressed in 2° cells. Besides acting as a direct repressor on *pcf* expression, HexA also represses production of the quorum sensing signal photopyrone D [13], and consequently indirectly inhibits *pcf* expression by silencing PluR-mediated quorum sensing in *P*. *luminescens*. A link between HexA and quorum sensing has also been found in the plant-pathogenic bacterium *Erwinia carotovora* ssp. *carotovora*, where HexA negatively regulates the production of the quorum sensing signal OHHL [N-(3-oxo-hexanoyl)-L-homoserine lactone], and thereby influences virulence [30,36]. In the plant-pathogenic bacterium *Pantoea stewartii* it was discovered that a high concentration of AHLs resulted in the deactivation of *lrhA* (*hexA* homologue) expression directly via the LuxR transcriptional regulator EsaR, which in turn is important for virulence against corn plants [37,38]. Thereby, it has been proposed that HexA is involved in a feedback loop on the quorum sensing network downstream of transcriptional regulator EsaR [39]. Since cell-clumping has also been proposed to contribute to the high virulence of *P*. *luminescens* and *Photorhabdus asymbiotica* [10,40], it is likely that HexA also indirectly influences pathogenicity of 2° cells. However, since pathogenicity of 2° cells has not been found to be attenuated in *P*. *temperata* [11] but 2° cells are not symbiotic any more, cell clumping might not only be important for pathogenicity, but also for symbiosis. This is supported by the fact that a Δ*pluR* deletion strain showed reduced reassociation with the nematodes (DJC and RH, unpublished results). Thereby, cell clumping might facilitate uptake of the bacteria by the nematodes.

HexA contains a C-terminal domain that is proposed to bind a putative, yet unidentified, molecule. SPR analyses and the respective association and dissociation rates with an overall affinity of 1.3 μM revealed that DNA-binding of HexA is not as stable as observed for other DNA-binding proteins. Therefore, the binding of a metabolite that might modulate binding affinity is likely. Since the metabolic state of the cell was proposed to somehow influence phenotypic switching [1], binding of a primary metabolite to HexA would be conceivable. Binding of metabolites to LysR-type regulators have been shown before. One example is ArgP of *E*. *coli*, which directly activates the transcription of the lysine transporter gene *lysP* and binds lysine to modulate DNA-binding affinity [41]. Since *P*. *luminescens* produces a huge set of secondary metabolites, whereby most of them are only present in 1° cells, binding of a compound of the primary metabolism to HexA might be likely. The LysR-type receptor RovM of *Yersinia pseudotuberculosis* regulates expression of the temperature-dependent virulence regulator gene *rovA* [42]. The secondary structure of RovM revealed that it binds small inducer molecules to modulate DNA-binding affinity [43]. We have preliminary tested different primary as well as secondary metabolites and their effect to modulate DNA-binding of HexA (data not shown). However, no putative ligand of HexA could yet be identified and therefore remains elusive.

**Fig 7:**
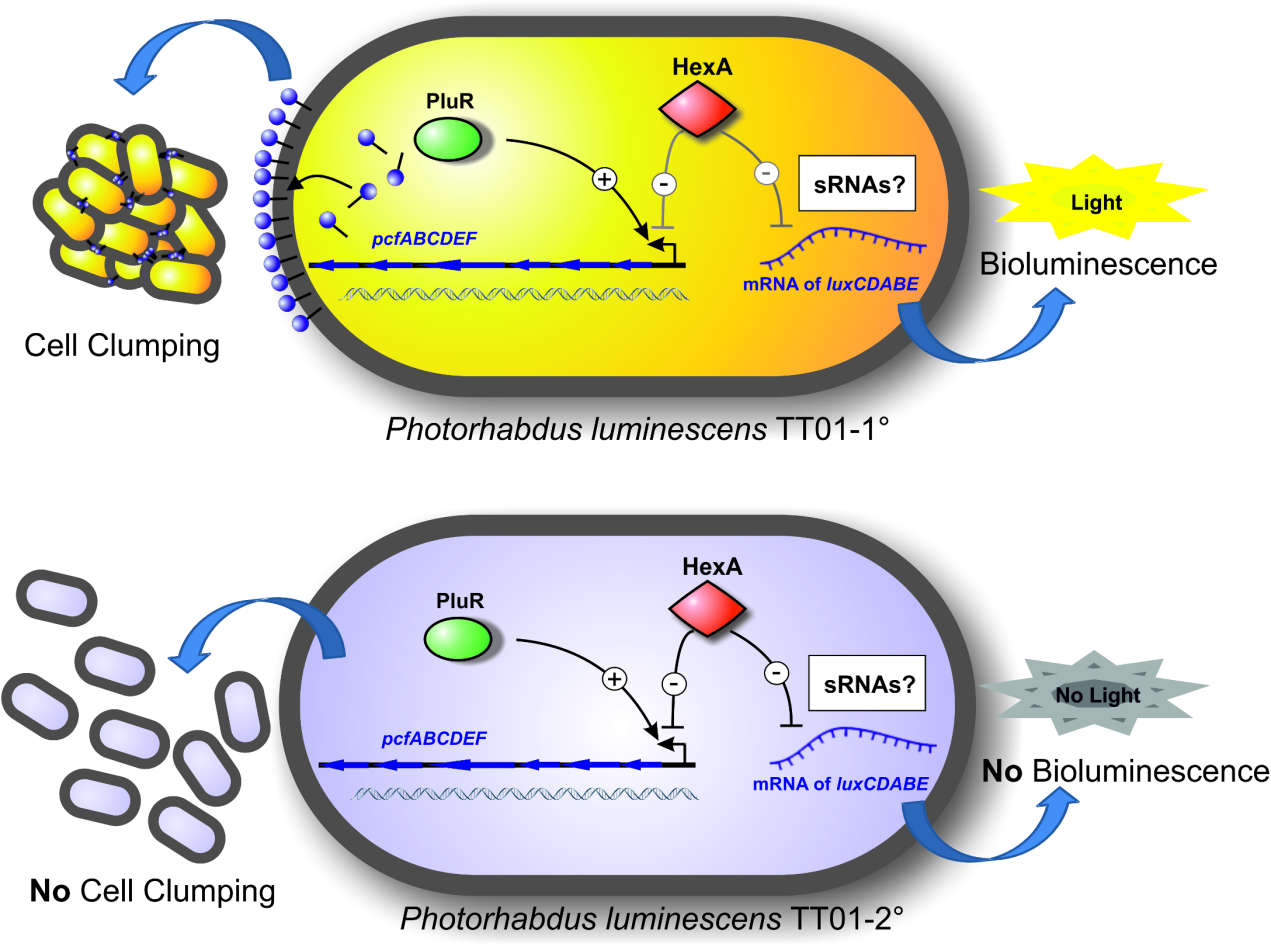
Model of the versatile role of HexA controlling 1° and 2° specific phenotypes in *P*. *luminescens*. HexA directly represses the promoter of the *pcfABCDEF* operon, which is responsible for the formation of cell clumps, and indirectly represses the translation of *luxCDABE*, presumably via sRNAs, and thereby diminishes light production in 2° cells.

Taken together we conclude that HexA is a versatile transcriptional regulator that directly but also indirectly represses 1° specific features in 2° cells of *P*. *luminescens* to regulate expression of a huge subset of different genes. However, it is unclear which role HexA plays in 1° cells, where it occurs at low levels. If HexA itself or its activity is regulated via other regulator(s), ligands or the involvement of Hfq remains to be determined (Fig 7).

## Supporting information

S1 File **Figure A. P**_***hexA***_ **activity in *P*. *luminescens* TT01-1°, TT01-2° and TT01-1°Δ*hexA* at the single cell level.** P_*hexA-*_*mCherry* activity in TT01-1°, TT01-2° and TT01-1°Δ*hexA* after 24 h of growth. The scale depicts 10 μM. Representative images from one of three independently performed experiments are shown.
**Figure B. Proteome analysis of *P*. *luminescens* TT01-1° and TT01-1°Δ*hexA*.** Cells were cultivated and harvested in exponential (A) and in the stationary phase (B). Cytosolic proteins were extracted and then subjected to 2D-PAGE. Gels were scanned, and compared for protein spots of different sizes. Proteins with enhanced production (□), with reduced production (▽) or overproduced (◊) in the Δ*hexA* mutant and proteins that were completely absent in the Δ*hexA* mutant (□) or in the wildtype (○) were analyzed via MALDI-TOF.
**Figure C. Cell clumping in *P*. *luminescens* TT01-1°, TT01-2° and TT01-1°Δ*hexA* after 7 days.** P_*pcfA*_ activity and cell clumping in TT01-1°, TT01-2° and TT01-1°Δ*hexA*. The scale depicts 10 μM. Representative images from one of three independently performed experiments are shown.
**Figure D. Effect of HexA on the P**_***pcfA***_ **activity in the heterologous systems of *E*. *coli* Δ*lrhA*.** In *E*. *coli* Δ*lrhA* the constructs pBAD24-P_*lac-*_*pluR*_P_*ara*_-*hexA* and pBBR-P_*pcfA*_-*lux* were tested. The expression of *pluR* was achieved via the addition of 1 mM IPTG and *hexA* expression was induced via the addition of 0.02 and 0.2% arabinose (Ara). The figure represents three biological replicates. All values are given in percentage, relative to the maximum *pluR* induction. The values were measured as Relative Light Unit [RLU] divided by OD_600nm_.
**Figure E. Investigation of an effect of HexA on the *lac* promoter and the *luxCDABE* operon**. The constructs pBAD24-P_*lac-*_*pluR*-P_*ara*_-*hexA* and pBBR-P_*lac*_*-lux* were tested in *E*. *coli*Δ*lrhA* and 1 mM IPTG was added. Expression of *hexA* was induced via the addition of 0.02–0.2% arabinose (Ara). The graph corresponds to measurements performed 3 hours after induction. The figures represent three biological replicates. All values are expressed in percentages, relative to the values of the *pluR* maximum induction upon addition of 1 mM IPTG.
**Figure F. Purification and biochemical investigation of HexA-6His.** Purification of HexA via Ni-NTA affinity chromatography. Left panel shows a Coomassie blue stained SDS gel; right panel shows a Western blot with αHexA antiserum. C = cytosolic fraction; W1 = washing fraction 1; W2 = washing fraction 2; E1 = elution fraction 1; E2 = elution fraction 2; E3 = elution fraction 3; E = pooled elution fraction (A). Gel filtration of purified HexA-6His (E) using Superdex 200 column (B). Size and molecular weight determination of “HexA” peak fraction (gel filtration) using Dynamic Light Scattering (DLS) (C). Stability measurement of HexA-6His in different buffers using a fluorescence-based thermal stability assay. Tm = melting temperature, TN = 50mM Tris/HCl pH 7.5, 200 mM NaCl; G = glycerol; β-MeOH = 2 mM β-mercaptoethanol (D).
Table A. Bacterial Strains.
Table B. Plasmids.
Table C. Oligonucleotides.
**Table D. Proteins with altered production in the proteome of TT01-1°Δ*hexA* compared to TT01-1°.** Differences in the cytosolic proteome were detected in the exponential (EX) and stationary (STAT) growth phase.
(PDF)
