# Supplementary material for: HexA is a versatile regulator involved in the control of phenotypic heterogeneity of Photorhabdus luminescens
Source: PLoS One. 2017 Apr 27;12(4):e0176535. doi: 10.1371/journal.pone.0176535 (PMC5407808; doi:10.1371/journal.pone.0176535)
Supplement: S1 File — Figure A. PhexA activity in P. luminescens TT01-1°, TT01-2° and TT01-1°ΔhexA at the single cell level. PhexA-mCherry activity in TT01-1°, TT01-2° and TT01-1°ΔhexA after 24 h of growth. The scale depicts 10 μM. Representative images from one of three independently performed experiments are shown. Figure B. Proteome analysis of P. luminescens TT01-1° and TT01-1°ΔhexA. Cells were cultivated and harvested in exponential (A) and in the stationary phase (B). Cytosolic proteins were extracted and then subjected to 2D-PAGE. Gels were scanned, and compared for protein spots of different sizes. Proteins with enhanced production (□), with reduced production (▽) or overproduced (◊) in the ΔhexA mutant and proteins that were completely absent in the ΔhexA mutant (□) or in the wildtype (○) were analyzed via MALDI-TOF. Figure C. Cell clumping in P. luminescens TT01-1°, TT01-2° and TT01-1°ΔhexA after 7 days. PpcfA activity and cell clumping in TT01-1°, TT01-2° and TT01-1°ΔhexA. The scale depicts 10 μM. Representative images from one of three independently performed experiments are shown. Figure D. Effect of HexA on the PpcfA activity in the heterologous systems of E. coli ΔlrhA. In E. coli ΔlrhA the constructs pBAD24-Plac-pluR_Para-hexA and pBBR-PpcfA-lux were tested. The expression of pluR was achieved via the addition of 1 mM IPTG and hexA expression was induced via the addition of 0.02 and 0.2% arabinose (Ara). The figure represents three biological replicates. All values are given in percentage, relative to the maximum pluR induction. The values were measured as Relative Light Unit [RLU] divided by OD600nm. Figure E. Investigation of an effect of HexA on the lac promoter and the luxCDABE operon. The constructs pBAD24-Plac-pluR-Para-hexA and pBBR-Plac-lux were tested in E. coliΔlrhA and 1 mM IPTG was added. Expression of hexA was induced via the addition of 0.02–0.2% arabinose (Ara). The graph corresponds to measurements performed 3 hours after induction. The figures represent t [file pone.0176535.s001.pdf]

Supporting Information

HexA is a versatile regulator involved in the control of phenotypic heterogeneity of *Photorhabdus luminescens*

Angela Langer[1], Adriana Moldovan[1], Christian Harmath[1], Susan A. Joyce[2], David J. Clarke[2] and Ralf Heermann[1]*

[1]*Bereich Mikrobiologie, Biozentrum Martinsried, Ludwig-Maximilians-Universität München, München, Germany*

[2]*School of Microbiology and Microbiome Institute, University College Cork, Cork, Ireland*

*Corresponding author
heermann@lmu.de

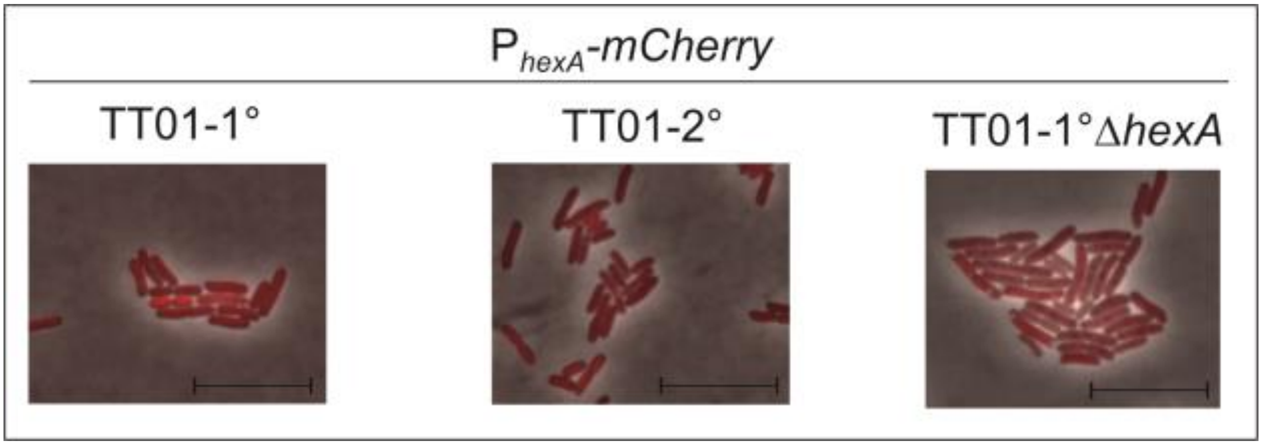

Figure A. P_{hexA} activity in *P. luminescens* TT01-1°, TT01-2° and TT01-1°Δ*hexA* at the single cell level. P_{hexA}-*mCherry* activity in TT01-1°, TT01-2° and TT01-1°Δ*hexA* after 24 h of growth. The scale depicts 10 µM. Representative images from one of three independently performed experiments are shown.

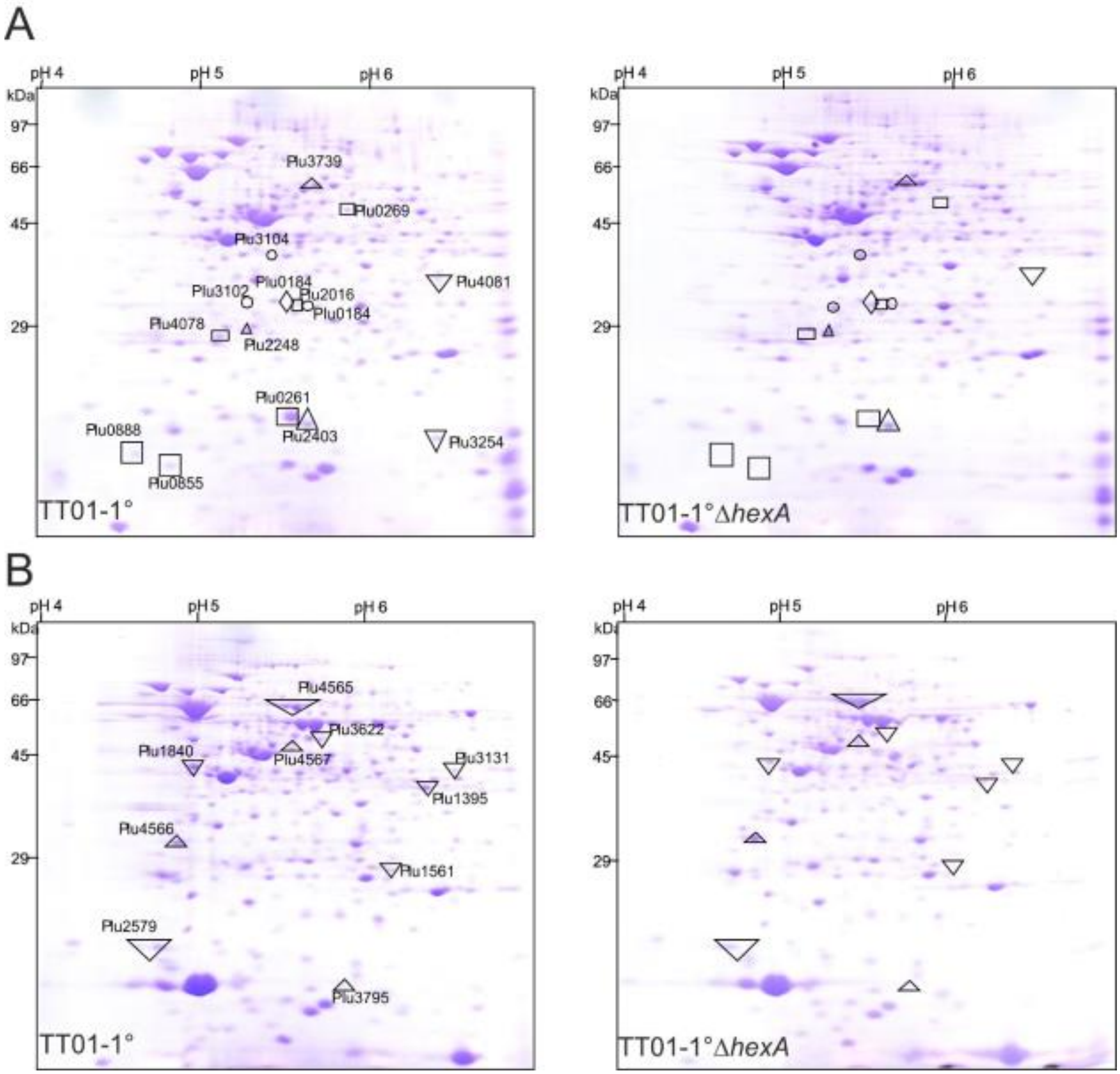

Figure B. Proteome analysis of *P. luminescens* TT01-1° and TT01-1°Δ*hexA*. Cells were cultivated and harvested in exponential (A) and in the stationary phase (B). Cytosolic proteins were extracted and then subjected to 2D-PAGE. Gels were scanned, and compared for protein spots of different sizes. Proteins with enhanced production (△), with reduced production (▽) or overproduced (◊) in the Δ*hexA* mutant and proteins that were completely absent in the Δ*hexA* mutant (□) or in the wildtype (○) were analyzed via MALDI-TOF.

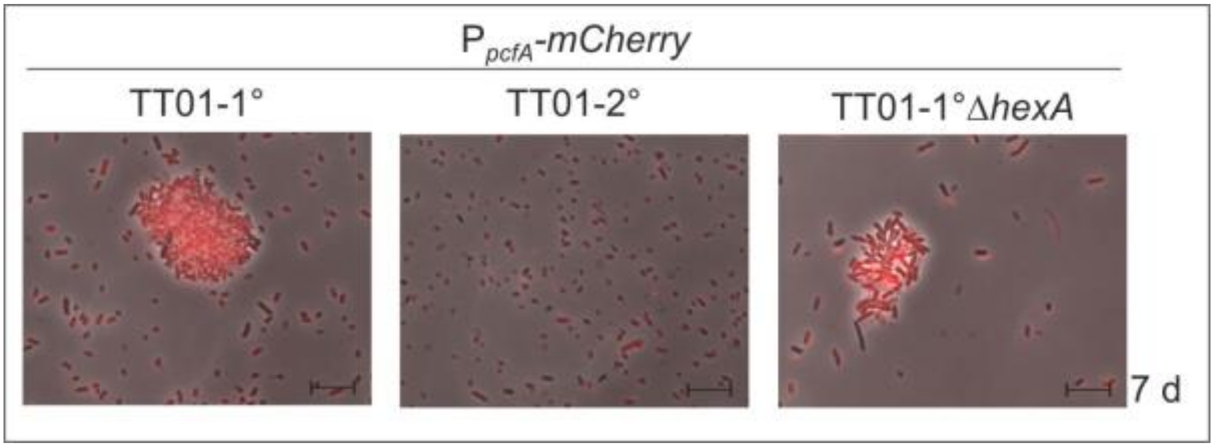

Figure C. Cell clumping in *P. luminescens* TT01-1°, TT01-2° and TT01-1°Δ*hexA* after 7 days. P_{pcfA} activity and cell clumping in TT01-1°, TT01-2° and TT01-1°Δ*hexA*. The scale depicts 10 µM. Representative images from one of three independently performed experiments are shown.

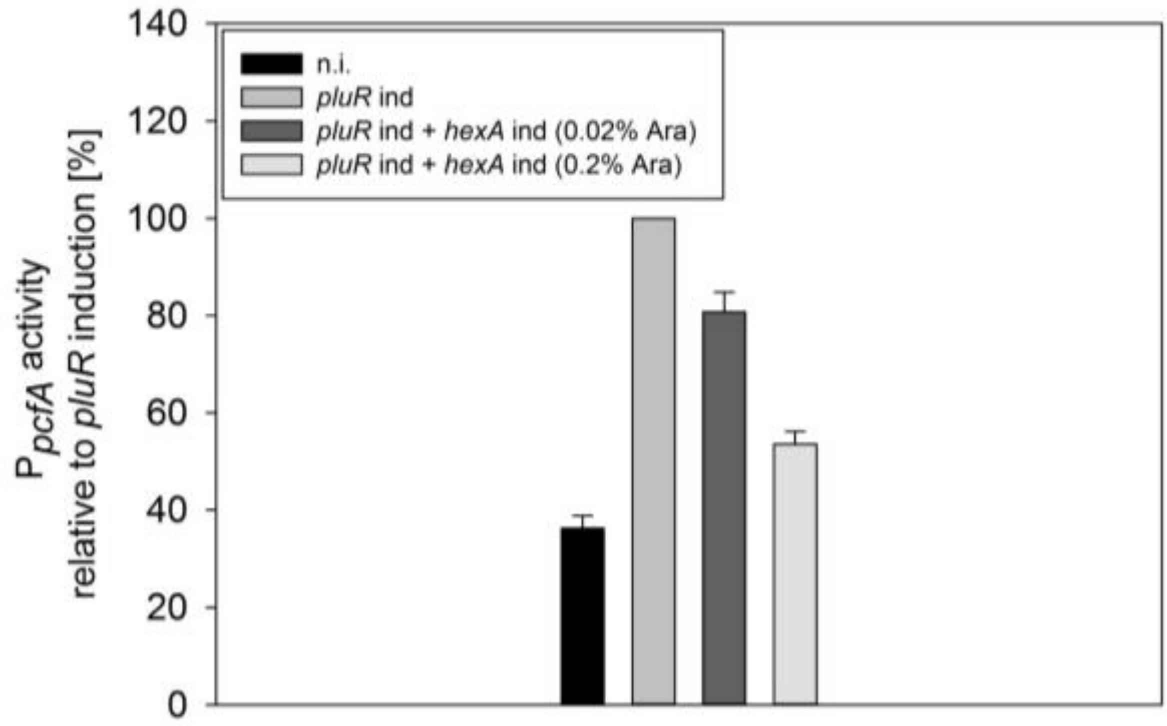

Figure D. Effect of HexA on the P_{pcfA} activity in the heterologous systems of *E. coli* Δ*lrhA*. In *E. coli* Δ*lrhA* the constructs pBAD24-P_{lac}-*pluR*_P_{ara}-*hexA* and pBBR-P_{pcfA}-*lux* were tested. The expression of *pluR* was achieved via the addition of 1 mM IPTG and *hexA* expression was induced via the addition of 0.02 and 0.2% arabinose (Ara). The figure represents three biological replicates. All values are given in percentage, relative to the maximum *pluR* induction. The values were measured as Relative Light Unit [RLU] divided by OD_{600nm}.

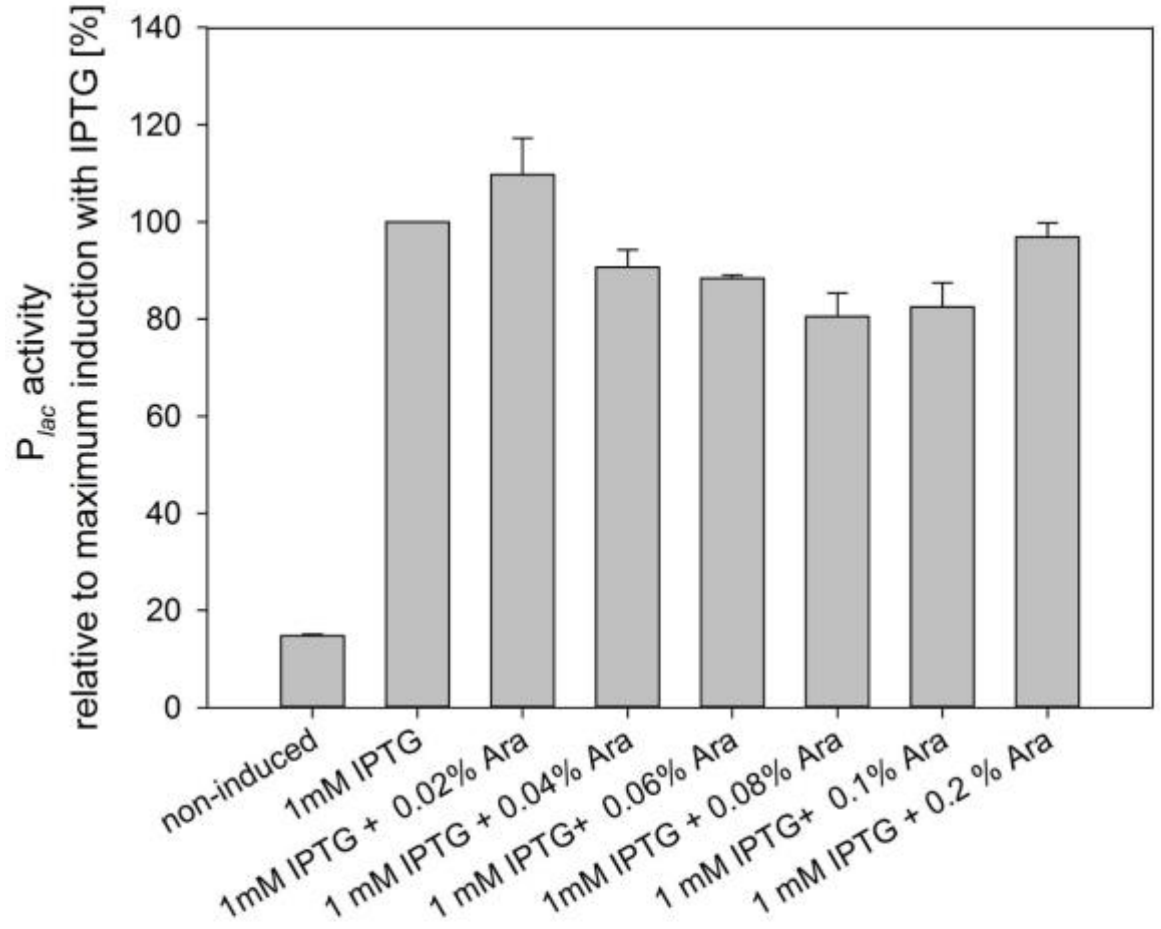

Figure E. Investigation of an effect of HexA on the *lac* promoter and the *luxCDABE* operon. The constructs pBAD24-P_{lac}-*pluR*-P_{ara}-*hexA* and pBBR-P_{lac}-*lux* were tested in *E. coli*Δ*lrhA* and 1 mM IPTG was added. Expression of *hexA* was induced via the addition of 0.02-0.2% arabinose (Ara). The graph corresponds to measurements performed 3 hours after induction. The figures represent three biological replicates. All values are expressed in percentages, relative to the values of the *pluR* maximum induction upon addition of 1 mM IPTG.

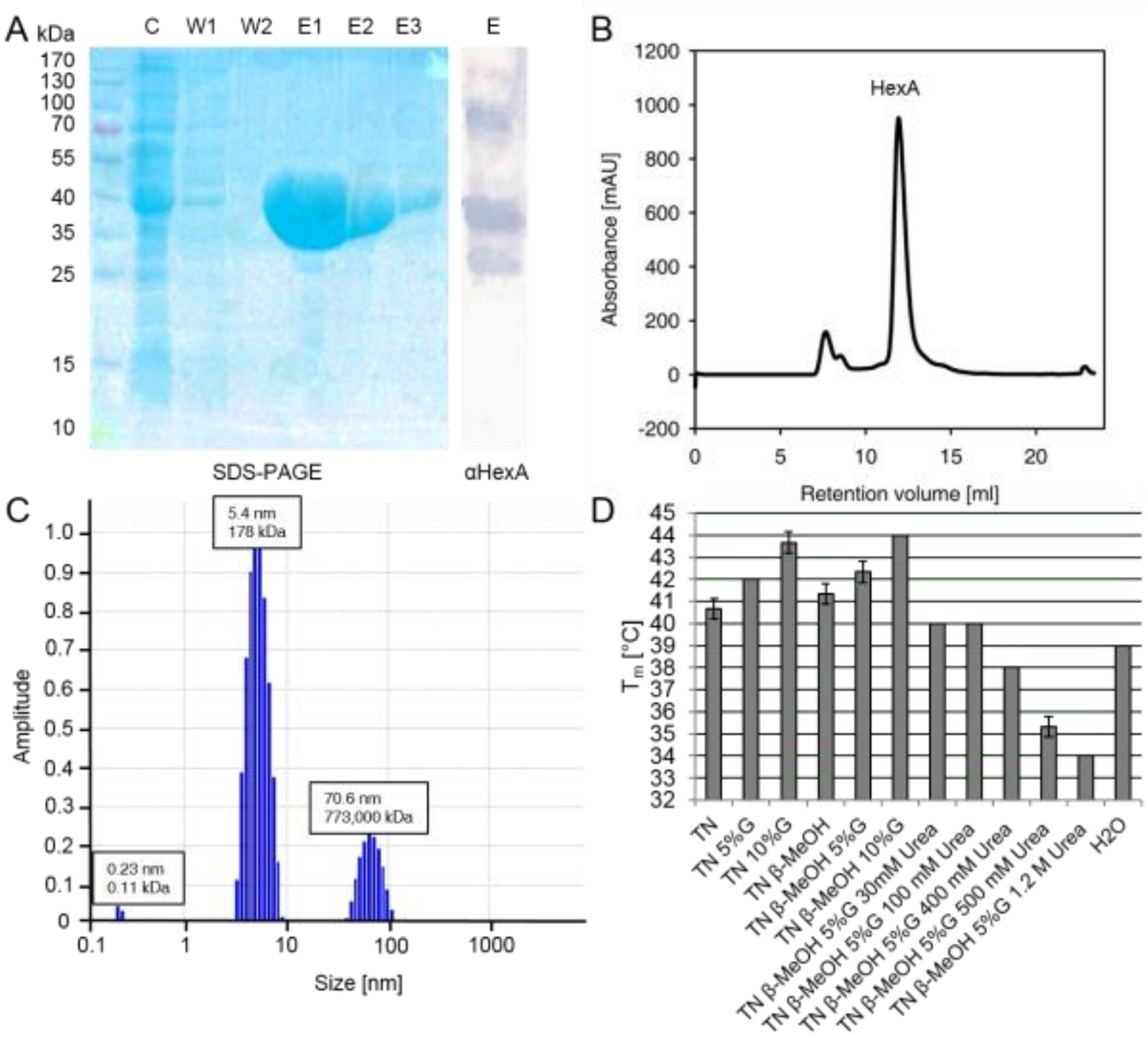

Figure F. Purification and biochemical investigation of HexA-6His. Purification of HexA via Ni-NTA affinity chromatography. Left panel shows a Coomassie blue stained SDS gel; right panel shows a Western blot with αHexA antiserum. C=cytosolic fraction; W1=washing fraction 1; W2=washing fraction 2; E1=elution fraction 1; E2=elution fraction 2; E3=elution fraction 3; E=pooled elution fraction (A). Gel filtration of purified HexA-6His (E) using Superdex 200 column (B). Size and molecular weight determination of "HexA" peak fraction (gel filtration) using Dynamic Light Scattering (DLS) (C). Stability measurement of HexA-6His in different buffers using a fluorescence-based thermal stability assay. Tm=melting temperature, TN=50mM Tris/HCl pH 7.5, 200 mM NaCl; G=glycerol; β-MeOH = 2 mM β-mercaptoethanol (D).

Table A. Bacterial Strains.

Bacterial Strain	Genotype	Reference
P. luminescens subsp. laumondi TT01-1°	Wild-type 1° variant, Rif^R	[1]
P. luminescens subsp. laumondi TT01-2°	Wild type 2° variant, Rif^R	Lab collection, Dr. David Clarke, University College Cork
P. luminescens TT01-1° Δ*hexA*	Wild-type 1° variant containing a deletion of *hexA (plu3090)*	Lab collection, Dr. David Clarke, University College Cork
P. luminescens TT01-1° P_{hexA}-*mCherry*	TT01-1° harboring P_{hexA}-*mCherry* reporter integrated at the *rpmE*/*glmS* site, Kan^R, $Gent^R$	[2]
P. luminescens TT01-2° P_{hexA}-*mCherry*	TT01-2° harboring P_{hexA}-*mCherry* reporter integrated at the *rpmE*/*glmS* site, Kan^R, $Gent^R$	This study
P. luminescens TT01-1° Δ*hexA* P_{hexA}-*mCherry*	TT01-1° Δ*hexA* harboring P_{hexA}-*mCherry* reporter integrated at the *rpmE*/*glmS* site, Kan^R, $Gent^R$	This study
P. luminescens TT01-1° P_{hexA}-*hexA-mCherry*	TT01-1° harboring P_{hexA}-*hexA-mCherry* reporter integrated at the *rpmE*/*glmS* site, Kan^R, $Gent^R$	This study
P. luminescens TT01-2° P_{hexA}-*hexA-mCherry*	TT01-2° harboring P_{hexA}-*hexA-mCherry* reporter integrated at the *rpmE*/*glmS* site, Kan^R, $Gent^R$	This study
P. luminescens TT01-1° Δ*hexA* P_{hexA}-*hexA-mCherry*	TT01-1° Δ*hexA* harboring P_{hexA}-*hexA-mCherry* reporter integrated at the *rpmE*/*glmS* site, Kan^R, $Gent^R$	This study
P. luminescens TT01-1° P_{luxC}-*mCherry*	TT01-1° harboring P_{luxC}-*mCherry* reporter integrated at the *rpmE*/*glmS* site, Kan^R, $Gent^R$	[2]
P. luminescens TT01-2° P_{luxC}-*mCherry*	TT01-2° harboring P_{luxC}-*mCherry* reporter integrated at the *rpmE*/*glmS* site, Kan^R, $Gent^R$	This study

P. luminescens TT01-1° Δ*hexA* P_{luxC}-*mCherry*	TT01-1° Δ*hexA* harboring P_{luxC}-*mCherry* reporter integrated at the *rpmE*/*glmS* site, Kan^R, $Gent^R$	This study
P. luminescens TT01-1° P_{hfq}-*mCherry*	TT01-1° harboring P_{hfq}-*mCherry* reporter integrated at the *rpmE*/*glmS* site, Kan^R, $Gent^R$	This study
P. luminescens TT01-2° P_{hfq}-*mCherry*	TT01-2° harboring P_{hfq}-*mCherry* reporter integrated at the *rpmE*/*glmS* site, Kan^R, $Gent^R$	This study
P. luminescens TT01-1° Δ*hexA* P_{hfq}-*mCherry*	TT01-1° Δ*hexA* harboring P_{hfq}-*mCherry* reporter integrated at the *rpmE*/*glmS* site, Kan^R, $Gent^R$	This study
P. luminescens TT01-1° P_{pcfA}-*mCherry*	TT01-1° harboring P_{pcfA}-*mCherry* reporter integrated at the *rpmE*/*glmS* site, Kan^R, $Gent^R$	This study
P. luminescens TT01-2° P_{pcfA}-*mCherry*	TT01-2° harboring P_{pcfA}-*mCherry* reporter integrated at the *rpmE*/*glmS* site, Kan^R, $Gent^R$	This study
P. luminescens TT01-1° Δ*hexA* P_{pcfA}-*mCherry*	TT01-1° *ΔhexA* harboring P_{pcfA}-*mCherry* reporter integrated at the *rpmE*/*glmS* site, Kan^R, $Gent^R$	This study
E. coli Dh5α λ*pir*	*recA1, gyrA (lacIZYA-argF) (80d lac [lacZ] M15) pir RK6*	[3]
E. coli S17-1 λpir	Tp^R Sm^R *recA*, *thi*, *pro*, *hsdR*-M+RP4: 2-Tc:Mu: Km Tn7 λpir	Biomedal S.L. Sevilla, Spain
E. coli ST18	*E. coli* S17 *λpir* Δ*hemA*	[4]
E. coli BL21 (DE3) Star	F^- *omp*T *hsd*S_B (r_B^- m_B^-) *gal dcm rne*131 (DE3)	Invitrogen
E. coli JW2284	Kan^R, BW25113 *lrhA::npt*	[5]
E. coli ΔlrhA	Removal of the *npt* cassette in *E. coli* JW2284 by P1 transduction	Dr. Sophie Brameyer, unpublished
Sh. oneidensis MR1 S79	Wild type isolate	[6]

Table B. Plasmids.

Plasmid	Genotype	Reference
pPINT-*mCherry*	Km^R, Gm^R and *mCherry* in pPINT	[2]
pPINT-P_{hexA}-*mCherry*	Km^R, Gm^R, *hexA* (*plu3090*) promoter upstream of *mCherry*	[2]
pPINT-P_{hexA}-*hexA*-*mCherry*	Km^R, Gm^R, *hexA* promoter upstream of *hexA (plu3090)*-*mCherry*	This study
pPINT-P_{luxC}-*mCherry*	Km^R, Gm^R, *luxC (plu2079)* promoter upstream of *mCherry*	[2]
pPINT-P_{hfq}-*mCherry*	Km^R, Gm^R, *hfq (plu4581)* promoter upstream of *mCherry*	This study
pPINT-P_{pcfA}-*mCherry*	Km^R, Gm^R, *pcfA (plu4568)* promoter upstream of *mCherry*	This study
pBAD24-*pluR*	Ap^R, *pluR* (*plu4562*) in pBAD24	[7]
pBAD24-*yehU*	Ap^R, *yehU*-6His in pBAD24 with a C-terminal HisTag	[8]
pBAD24-*hexA*	Ap^R, *hexA*-6His (*plu3090*) in pBAD24 with a C-terminal HisTag	This study
pCOLA-*ppyS*-His-*pluR*	Km^R, *ppyS (plu4844*) and 6His-*pluR* (*plu4562*) in pCOLA, IPTG inducible	Dr. Sophie Brameyer, unpublished
pBAD24-P_{ara}-*pluR*_P_{lac}-*hexA*	Ap^R, *pluR* (*plu4562*) under the control of an arabinose inducible promoter, *hexA* (*plu3090*) under the control of an IPTG inducible promoter	This study
pBAD24-P_{lac}-*pluR*_P_{ara}-*hexA*	Ap^R, *pluR* (*plu4562*) under the control of an IPTG inducible promoter, *hexA*	This study

	(*plu3090*) under control of an arabinose inducible promoter	
pBBR1-P_{pcfA}-*lux*	Gm^R, l*uxCDABE* under the control of the *pcfA* (*plu4568*) promoter	[7]
pBBR1-P_{pcfA}-s1-*lux*	Gm^R, *luxCDABE* under the control of the truncated promoter construct P_{pcfA}-S1	Dr. Sophie Brameyer, unpublished
pBBR-P_{pcfA}-s2-*lux*	Gm^R, *luxCDABE* under the control of the truncated promoter construct P_{pcfA}-S2	Dr. Sophie Brameyer, unpublished
pBBR-P_{lac}-*lux*	Gm^R, *luxCDABE* under the control of the *lac* promoter	This study
pACYC-Duet1	Cm^R, Expression vector, IPTG inducible	Novagen®
pACYC-*hexA*	Cm^R, *hexA* (*plu3090*) in pACYC-Duet1	This study
pACYC-P_{lac}-*hexA*_P_{ara}-*pluR*	Cm^R, *pluR* (*plu4562*) under the control of an arabinose inducible promoter, *hexA* (*plu3090*) under the control of an IPTG inducible promoter	This study
pEYFP	Ap^R, *lac*-promoter upstream of *eYFP*	Takara-Clonetech, Saint-Germain-en-Laye, France)
pD132	Cm^R, ori R6K, oriT RK2, *sacB*	[9]
pDS-*hexA*	Flanking regions of *hexA* (*plu3090*) in pD132	This study

Table C. Oligonucleotides.

Primer name	Sequence (5`-3`)
PhexA-BamHI_fwd	GCTGGATCCTCTTACCTTATCTTGGTAAA
hexA-XmaI _rev	GCTCCCGGGCTCATCAATAATATCGTCATCATCA
Phfq-NheI_fwd	GCGGCTAGCTCACTGAACTGACTACATTG
Phfq-BamHI_rev	GCTGGATCCTCTATATTTTCCTTATTTTGTT
PpcfA-NheI_fwd	AATGGAGCTAGCAGCAGAATTCGGGTTAGTTATCTATGC
PpcfA-XmaI_rev	ACTAAGCCCGGGACCAGCTTTATCCCTTATGTC
check-mcherry_ins_fwd	CTGGTTTCATAATTTCGCC
check-mcherry-ins_rev	GGCCTTCCTTCTCCTTCAC
check-rpmE_fwd	CTCCCAAATAAAGTTTAGG
check-glmS_rev	GTACGTGAATCTGATTTTG
oriT_fwd	CAGGGTTATGCAGCGGAAA
gmRpNPTS_fwd	GATAAGCTGTCAAACATGAGAGTAGCGTATGCGCTCAC
Plac(h)_fwd	ATTGCATTTATCATGGTATATCTCCTTATTAAA
PlacI-SalI_rev	GCTGTCGACTCACTGCCCGCTTTCCAGTC
hexA_fwd	ATGATAAATGCAAATCGTC
hexA-PstI-rev	GCTCTGCAGTTACTCATCAATAATATCG
pBAD24_seq_fwd	GCCGTCACTGCGTCTTTTACTGG
pBAD24_seq_rev	CGCTACGGCGTTTCACTTCTG
hexA-EcoRI_fwd	GCTGAATTCATGATAAATGCAAATCGTCC
hexA-NdeI_rev	GCGCATATGCTCATCAATAATATCGTCATCATC
Plac-PluR_fwd	TCTTCAAAGCTTGCGGCCGCATAATG
PluR-PstI_rev	GCGCTGCAGGTTATATGATTAGATTATATGCTATTGC
lacI_fwd	CAAGCTTTGAAGATCGAATGGCGCAAAACCTT
lacI-SalI_rev	GCTGTCGACTCACTGCCCGCTTTCCAGTC

check-PlachexA_fwd	CTACCAGAGAAGTTGAAGT
hexA-NcoI_fwd	GCTCCATGGATGATAAATGCAAATCGTCC
hexA-SalI_rev	GCGGTCGACTTACTCATCAATAATATC
check-pACYC_fwd	ATTCACCACCCTGAATTGA
check-pACYC_rev	CTAGTTATTGCTCAGCGGT
araCPluR_fwd	GCGCATATGACTCCGTCAAGCCGTCAA
pluR-XhoI_rev	TAGCCCTCGAGCTGTGATGATGATGATGATGATGATGATGATG
	ACGACCTTCGATATGGCCGCTTATATGATTAGATTATATGC
PpcfA-Btn_fwd	TATTTGTCTTTATAATGATAAT
PpcfA_rev	ACCAGCTTTATCCCTTATGTC
sacB-Btn_fwd	GCAGAAGTTTTTGACTTTCTTG
sacB_rev	ACATCTGACGGAAAAATCCGT
Plac-NheI_fwd	GCGGCTAGCGCGCAACGCAATTAATGTG
Plac-BamHI_rev	CGCGGATCCAGCTGTTTCCTGTGTGAAA
check-pBBR-Plac_fwd	CCGTCGTATTAAAGAGGGG
FA_hexA_fwd	GAATTGTTGTTGTTTTTTA
FA_hexA_rev	CATTGTTTATTCATCACTTT
FB_hexA_fwd	TAATATCTGAAACACTTCTC
FB_hexA_rev	AATCAATGATTGATGGAGTG

Table D. Proteins with altered production in the proteome of TT01-1°Δ*hexA* compared to TT01-1°. Differences in the cytosolic proteome were detected in the exponential (EX) and stationary (STAT) growth phase.

Protein	Putative function	Growth phase	Δ*hexA*/wild-type
Plu0184 (CpmC)	Role in Carbapenem biosynthesis		+4.2
Plu0261	Similiarities with type 1 fimbrial protein precursor	EX	n.d. in Δ*hexA*
Plu0269	Unknown, hypothetical secreted protein	EX	n.d. in Δ*hexA*
Plu0885	Pyocin S3 protein, „killer protein“	EX	n.d. in Δ*hexA*
Plu0888	Colicin/Pyocin protein, „killer protein“	EX	n.d. in Δ*hexA*
Plu1395	Cystein Synthase A	STAT	-1.7
Plu1561	Ca^{2+}-dependent cell adhesion molecule	STAT	-2.6
Plu1840	unknown	STAT	-1.6
Plu2016	PAS4-LuxR regulator	EX	n.d. in Δ*hexA*
Plu2248	Carbonic anhydrase	EX	+4.0
Plu3102	methyltransferase	EX	n.d. in WT
Plu3104	unknown	EX	n.d. in WT
Plu3110 (ArgM)	Succinylornithine transaminase	STAT	-1.4
Plu3254	Hcp family T6SS protein CtsH1	EX	-2.7
Plu3622 (AceF)	dihydrolipoamide acetyltransferase; pyruvate dehydrogenase subunit E2	STAT	-2.2
Plu3739 (AldB)	Aldehyde Dehydrogenase B	EXP	+1.8
Plu3795	unknown	STAT	+2.4
Plu4078	Dimethylmenaquinone methyltransferase	EXP	n.d. in Δ*hexA*
Plu4081	Putative aldolase		-3.2
Plu4565	Cysteine synthase	STAT	+2.0

(PcfA)			
Plu4567 (PcfB)	Ariginosuccinate synthase	STAT	+2.0
Plu4566 (PcfC)	Glycine amidino transferase	STAT	+2.1

References

1. Duchaud E, Rusniok C, Frangeul L, Buchrieser C, Givaudan A et al. (2003) The genome sequence of the entomopathogenic bacterium *Photorhabdus luminescens.* Nature Biotechnol 21 (11): 1307–1313.
2. Glaeser A, Heermann R (2015) A novel tool for stable genomic reporter gene integration to analyze heterogeneity in *Photorhabdus luminescens* at the single-cell level. BioTechniques 59 (2): 74–81.
3. Miller VL, Mekalanos JJ (1988) A novel suicide vector and its use in construction of insertion mutations: osmoregulation of outer membrane proteins and virulence determinants in Vibrio cholerae requires toxR. J Bacteriol 170 (6): 2575–2583.
4. Thoma S, Schobert M (2009) An improved *Escherichia coli* donor strain for diparental mating. FEMS Microbiol Lett 294 (2): 127–132.
5. Baba T, Ara T, Hasegawa M, Takai Y, Okumura Y et al. (2006) Construction of *Escherichia coli* K-12 in-frame, single-gene knockout mutants: the Keio collection. Mol Syst Biol 2: 2006.0008.
6. Venkateswaran K, Moser DP, Dollhopf ME, Lies DP, Saffarini DA et al. (1999) Polyphasic taxonomy of the genus Shewanella and description of *Shewanella oneidensis* sp. nov. Int J Syst Evol Microbiol 49 Pt 2: 705–724.
7. Brachmann AO, Brameyer S, Kresovic D, Hitkova I, Kopp Y et al. (2013) Pyrones as bacterial signaling molecules. Nat Chem Biol 9 (9): 573–578.
8. Fried L, Behr S, Jung K (2012) First insights into the unexplored two-component system YehU/YehT in *Escherichia coli.* J Bacteriol 194 (16): 4272–4284.
9. Philippe N, Alcaraz J, Coursange E, Geiselmann J, Schneider D (2004) Improvement of pCVD442, a suicide plasmid for gene allele exchange in bacteria. Plasmid 51 (3): 246–255.
